# Global Development Assistance for Adolescent Health From 2003 to 2015

**DOI:** 10.1001/jamanetworkopen.2018.1072

**Published:** 2018-08-10

**Authors:** Zhihui Li, Mingqiang Li, George C. Patton, Chunling Lu

**Affiliations:** 1 Department of Global Health and Population, Harvard T. H. Chan School of Public Health, Boston, Massachusetts; 2 Centre for Adolescent Health, Murdoch Children’s Research Institute and University of Melbourne, Parkville, Victoria, Australia; 3 Division of Global Health Equity, Brigham and Women’s Hospital and Harvard Medical School, Boston, Massachusetts; 4 Department of Science and Technology–National Research Foundation Center of Excellence in Human Development, University of Witwatersrand, Johannesburg, South Africa

## Abstract

**Importance:**

Growth in financing has underpinned progress in most areas of health. Adolescent health has recently become a global priority, with inclusion in the Global Strategy for Women’s, Children’s and Adolescents’ Health, but little is known about patterns of financing and development assistance for adolescent health (DAAH).

**Objective:**

To provide estimates of DAAH at global, regional, and country levels.

**Design, Setting, and Participants:**

In this quality improvement study, data from the Creditor Reporting System were used to estimate flows of total DAAH and per-adolescent DAAH and to assess its distribution by donors, regions, and countries and the leading causes of burden of disease (ie, disability-adjusted life-years) in 132 developing countries between January 1, 2003, and December 31, 2015. Through use of a key word search and various funding allocation methods, 2 sets of estimates were produced: adolescent-targeted DAAH that included disbursements to projects with a primary adolescent health target and adolescent-inclusive DAAH that included disbursements to projects with either a primary or partial adolescent health target, as well as projects that could benefit adolescent health but did not include age-related key words.

**Main Outcomes and Measures:**

Estimates of DAAH distinguishing between adolescent-targeted and adolescent-inclusive DAAH.

**Results:**

There were 19 921 projects in 132 countries in the adolescent-targeted estimation between 2003 and 2015, with a total funding amount of $3634.6 million, accounting for 1.6% of total development assistance for health. The top 5 donors (Global Fund to Fight AIDS, Tuberculosis and Malaria, $806.8 million; United Nations Population Fund, $401.3 million; United States, $389.9 million; United Kingdom, $251.8 million; and International Development Association, $218.6 million) together provided 56.9% of all adolescent-targeted DAAH. Sub-Saharan Africa received the largest cumulative DAAH per adolescent ($5.37) during the period. In 2015, among the 10 leading causes of disability-adjusted life-years, HIV and AIDS received the largest DAAH, followed by interpersonal violence, tuberculosis, and diarrheal diseases. Other leading causes, including road injuries and depressive disorders, received few disbursements, especially among the low-income countries.

**Conclusions and Relevance:**

Despite an increasing rate, DAAH composed a small proportion of total development assistance for health, suggesting that adolescent health has gained little donor attention. Moreover, recent allocations of DAAH have not aligned well with either the burden of disease or the areas where the benefits of investment are likely to be high.

## Key Points

**Questions:**

What have been the trends in worldwide development assistance for adolescent health between 2003 and 2015, and how well has it aligned with disease burden?

**Findings:**

This quality improvement study found that development assistance for adolescent health in 132 countries cumulatively accounted for only 1.6% of development health assistance. The largest amount of development assistance for adolescent health went to HIV and AIDS, while other leading causes of disease burden among adolescents, including anemia, road injuries, and depressive disorders, have been largely overlooked by donors.

**Meaning:**

Taking adolescent health to scale in the Global Strategy for Women’s, Children’s and Adolescents’ Health will require greater allocation of development funds and better targeting toward major causes of disease burden.

## Teaser

This quality improvement study estimates flows of total development assistance for adolescent health and per-adolescent development assistance for adolescent health and assesses its distribution by donors, regions, and countries and the leading causes of burden of disease in 132 developing countries.

## Introduction

Adolescence is a phase of rapid physical, cognitive, and emotional growth, in which future patterns of adult health are established.^1,2^ Today, 1.8 billion 10- to 24-year-olds, one-fourth of the world’s population, are central to major agendas in global health and international development, including noncommunicable diseases, injury, infectious diseases (including HIV), and mental health. Given that adolescents are the next generation to become parents, they are increasingly seen as central to the early life development of the next generation.^1,2,3,4,5^

Policy and research interest in adolescent health has been comparatively recent.^6^ However, the growing recognition of the significance of growth health and development during adolescence has now been recognized with the inclusion of adolescents in the Every Woman Every Child agenda through the Global Strategy for Women’s, Children’s and Adolescents’ Health.^7^ Development assistance for health (DAH) plays a major role in financing health care in developing countries where service systems and human capacity is generally limited.^8,9,10,11,12,13,14,15^

We assessed development assistance for adolescent health (DAAH) with adolescent-targeted and adolescent-inclusive estimates for 132 developing countries between 2003 and 2015. We provide an analysis of total DAAH and per-adolescent DAAH flows by donors, recipient regions, and countries, as well as the leading causes of disability-adjusted life-years (DALYs) in adolescents.

## Methods

### Data Sources

The aid data used in this study were extracted in July 2017 from the publicly accessible Creditor Reporting System (CRS) database for January 1, 2003, to December 31, 2015. Following the CRS recommendation, we did not use the disbursements data before 2003 owing to the large proportion of missing data.^16^ The CRS provides detailed aid activities, at the project level, reported directly by the following 4 types of donors: 30 members of the Development Assistance Committee, 32 multilateral organizations (eg, United Nations and World Bank), 20 non–Development Assistance Committee countries (eg, United Arab Emirates), and 1 private donor (Bill & Melinda Gates Foundation).^16^ The donors included in the CRS report are listed in eTable 1 in the Supplement. Our DAH estimate in 2015, derived from the CRS, is about 66% of the DAH estimate produced by Dieleman et al,^12^ which used data from 35 sources, including the CRS. The DAH derived from the CRS is presented in eTable 16 in the Supplement. This study used publicly accessible and secondary aid data at the aggregate level and therefore qualified for exemption of institutional review board approval from Harvard University. This study followed the Standards for Quality Improvement Reporting Excellence (SQUIRE) reporting guideline.

The projects are implemented in 147 low-income countries (LICs) and middle-income countries. All disbursement data were converted to constant 2015 US dollars with the donor-specific development assistance committee deflators.^17^ We excluded 15 recipient countries that either are high-income countries or do not have available data on adolescent populations because we cannot calculate DAAH per adolescent for these countries. We finally kept 132 countries in the analysis (eTable 2 in the Supplement). Annual data on the adolescent population were derived from data provided by the United Nations Population Division.^18^ Because the United Nations Population Division data by age group are reported every 5 years, we imputed the years between, assuming that the population was growing at the same rate each year.

### Defining and Identifying Projects for Adolescent Health

Adolescence is defined by the World Health Organization as the ages between 10 and 19 years.^19^ We extended this definition to all youths aged 10 to 24 years because our data examination shows that many aid projects (6132 of 19 921 [30.8%]) specify that they target populations between the ages 15 and 24 years, which cover those aged 15 to 19 years. The definition of adolescents as those aged 10 to 24 years has also been adopted in previous studies on adolescent health; this wider age group has sometimes been referred to as *young people*.^1^

The CRS data have no variable indicating projects targeting adolescent health. To identify relevant projects, we followed previous practices^8,9,11,20^ and identified DAAH projects with the combination of 2 strategies. The first strategy identified projects using a key word search.^11,21^ We generated key words for the related age groups (eg, “adolescent,” “teen,” “youth,” “aged 10-14” [eTable 3 in the Supplement]) and conducted key word searches on 3 variables in the CRS that provide descriptions of a project’s main objectives and targets: project title and short and long description of a project. The key word search was performed in the sectors listed in Table 1 (eg, health, education, government and civil society, and humanitarian aid).^22^ This strategy allowed us to obtain the projects that either exclusively or partially target adolescent health or well-being, as stated in their project descriptions.

**Table 1.** Frequency and the Most Common Themes of Projects for Development Assistance for Adolescent Health by Sector

Sector Name^a^	Sector Code	Frequency (%) of Total Project^b^	Examples
Education			
Basic education	112	2073 (10.4)	Sexual and reproductive health education for youths
Secondary education	113	529 (2.7)	Health education for young teens
Postsecondary education	114	268 (1.3)	Treat disruptive behavior and attention disorder of adolescence
Education level unspecified	111	268 (1.3)	Nutritional support for school-aged adolescents
Health			
General	121	556 (2.8)	Early adolescent motherhood in developing countries
Basic health	122	1056 (5.3)	Primary health care for adolescents
Population and reproductive health	130	9964 (50.0)	Adolescent sexual and reproductive health and HIV prevention
Water and sanitation	140	171 (0.9)	Provide safe drinking water for youths and young women
Government and civil society			
General	151	1832 (9.2)	Empower the right of young women to physical, emotional, and sexual health
Conflict, peace, and security	152	490 (2.5)	Improve the health of conflict-affected young people
Other social infrastructure and services	160	1385 (7.0)	Address youth HIV and AIDS and violence prevention
Economic infrastructure and services			
Transport and storage	210	22 (0.1)	Road safety for youths
Communications	220	121 (0.6)	Digital repository and online consultation system on health problems in youths and adolescents
Multisector and cross-cutting^c^	430	734 (3.7)	Implementation phase for the Adolescent Girls Initiative in Kenya to support 10 000 adolescent girls
Humanitarian aid			
Emergency response	720	93 (0.5)	Emergency response to sexual and gender-based violence
Reconstruction relief and rehabilitation	730	15 (0.08)	Give a perspective to traumatized young people through project work
Disaster prevention and preparedness	740	31 (0.2)	Raising youth awareness of health-affected disasters in school program
Unallocated and unspecified^d^	998	313 (1.6)	Education on STIs, HIV and AIDS, and reproductive health

Abbreviation: STIs, sexually transmitted infections.

^a^ See the Ministry for Foreign Affairs of Finland website for the definition of each sector category.^22^

^b^ For 19 921 projects.

^c^ Multisector and cross-cutting projects are environment activities not allocable to any specific sectors.

^d^ Unallocated and unspecified projects include those without specified sectors and aiming to promote development awareness.

The second strategy took into account the fact that projects that do not have age-related key words in their description, such as those for health system strengthening, could also benefit adolescents. To allocate part of the funds of these projects to adolescent health, we followed previous practices used by the Countdown group^10,11^ and the SQUIRE reporting guideline for quality improvement studies,^23^ and conducted the imputation for the projects with primary purpose listed in eTable 4 in the Supplement (eg, general budget support, family planning, health education, or infectious diseases control). The imputation was processed in the following ways: For general budget support, the allocation factor was generated by multiplying the country-specific proportion of government spending on health by the proportion of population between 10 and 24 years of age in that year in the country.^18,24^ For the projects related to family planning and reproductive health, we allocated the funds for adolescent health using the proportion of the population between 15 and 24 years of age in that year within the population between 15 and 49 years of age to reflect women’s active reproductive age.^25^ For other funding categories, we allocated the funds for adolescent health according to the country-specific proportion of the population between 10 and 24 years of age in that year in the total population (eTable 4 in the Supplement).

We translated all key words into 8 major languages: Spanish, French, Portuguese, Italian, Dutch, German, Norwegian, and Swedish. After conducting a key word search, we investigated how many projects that were not related to DAAH were falsely coded as DAAH projects when using the key word search. We manually reviewed the 3 variables for the selected projects and corrected for misspecification. For example, we excluded projects on “environmental health” that were captured with the key words of “mental health.” We excluded 22 103 projects that were falsely coded as DAAH and kept 276 002 projects.

Previous studies observed that a key word search strategy could lead to missing some projects for DAAH owing to imperfect sensitivity of the strategy.^20,26,27,28^ We investigated the percentage of projects for DAAH that were not captured by a key word search. To do so, we randomly selected 10.0% of all projects from the health sector (3268 of 32 680 projects) and 2.0% from other sectors (3871 of 193 541 projects) in 2015, and we applied the key word search and manually coded the selected projects separately. We then compared the results from the 2 methods and found that the key word searches missed about 11 of 7139 DAAH projects (0.15%).

Projects identified by the 2 strategies were allocated to adolescent-targeted and adolescent-inclusive categories. We defined the adolescent-targeted DAAH as the aid for projects with the primary objective (19 921 projects) of preventing diseases and maintaining, restoring, and improving the health of those aged 10 to 24 years. The adolescent-inclusive DAAH was the sum of the following 3 components: adolescent-targeted DAAH; projects that partially targeted adolescent health but also benefited members of other age groups, such as children; and projects that benefited adolescent health but did not specifically mention that they either exclusively or partially targeted adolescent health, such as family planning projects. Following earlier studies,^10,11,12,14,20^ we used actual disbursements to estimate donors’ contributions to DAAH because disbursements reflect the actual amount of resources delivered.

### Analyzing the Projects for DAAH

Using both the adolescent-targeted and adolescent-inclusive estimates, we tracked levels and trends of DAAH spending between 2003 and 2015 in total and per adolescent. We also identified the top 10 donors during the study period and disaggregated DAAH by recipient regions and countries. When estimating at regional or country levels, we excluded projects not allocable to a specific region or country.

We tracked the DAAH and specifically targeted the top 10 DALYs of adolescents estimated by the Institute for Health Metrics and Evaluation, including skin and subcutaneous diseases, road injuries, HIV and AIDS, iron-deficiency anemia, self-harm, interpersonal violence, depressive disorders, low back and neck pain, diarrheal diseases, and tuberculosis.^29^ The definitions and the level of leading DALYs are presented in eTable 5 in the Supplement.

To identify the projects targeting the 10 leading DALYs, we used a combination of age-related key words (eTable 3 in the Supplement) and target-specific key words for searching. The target-specific key words for the 10 leading DALYs of adolescent health were derived from previous studies^9,10,11,20^ and our review of scientific journals and reports. The key words for each of these areas are presented in eTables 6 to 15 in the Supplement. For projects with multiple purposes (eg, both HIV and AIDS and tuberculosis), we followed previous studies^8,20^ and included projects and estimations of both health focuses.

## Results

### Number of Adolescent-Targeted DAAH Projects in Each Sector

There were 19 921 projects targeting adolescent health in the adolescent-targeted estimation. Table 1 shows that 11 576 projects (58.1%) were allocated to the health service sector (including general health, basic health, and population and reproductive health) and that the remaining 8345 projects (41.9%) were allocated to nonhealth sectors. Population and reproductive health received the largest number of projects (9964 [50.0%]), with the most common themes being adolescent sexual and reproductive health, as well as HIV prevention. The education sector received 3138 projects (15.8%), with health education and school-based health interventions (eg, nutrition or behavioral intervention) the most common themes. Government and civil society received 2322 projects (11.7%), with the most common themes being empowering young women and improving the health of young people affected by armed conflict. The social infrastructure and services sector received 1385 projects (7.0%), with the most common themes addressing youth HIV and AIDS and prevention of youth violence. All other sectors together, including water and sanitation, economic infrastructure and services, multisector and cross-cutting, humanitarian aid, and unallocated or unspecified, had 1500 projects (7.5%).

### Estimates of Total Aid Disbursed to Adolescent-Targeted DAAH

**Figure 1.**
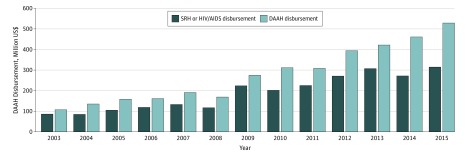
Trends in Annual Adolescent-Targeted Development Assistance for Adolescent Health (DAAH) and Annual Sexual and Reproductive Health (SRH) and HIV and AIDS Disbursement, 2003-2015 All disbursement data were converted to constant 2015 US dollars.

Between 2003 and 2015, $3634.6 million was disbursed to DAAH, accounting for 1.6% of the total DAH of $222 974 million. Figure 1 shows that DAAH increased approximately 5-fold, from $109.7 million in 2003 to $528.5 million in 2015. Disbursement for sexual and reproductive health and HIV and AIDS increased more than 3.6 times, from $87.4 million in 2003 to $315.4 million in 2015. Sexual and reproductive health and HIV and AIDS accounted for 59.0% ($271.3 million of $461.2 million) to 81.5% ($224.8 million of $276.0 million) of total DAAH in each year studied and cumulatively accounted for 68.0% ($2473.0 million of $3634.6 million) of total DAAH disbursed between 2003 and 2015. As a percentage of total DAH, DAAH increased from 1.3% ($109.7 million of $8566.5 million) in 2003 to 2.2% ($528.5 million of $24 008.5 million) in 2015 (eFigure 1 and eTable 16 in the Supplement). Estimates of adolescent-inclusive DAAH disbursements show a similar increasing trend during the study period (eFigure 2 in the Supplement).

### Estimates of Total Aid Disbursements to Adolescent-Targeted DAAH by Donors

The top 10 donors providing the largest cumulative funding between 2003 and 2015 are presented in eFigure 3 in the Supplement. The top 5 donors (Global Fund to Fight AIDS, Tuberculosis and Malaria, $806.8 million; United Nations Population Fund, $401.3 million; United States, $389.9 million; United Kingdom, $251.8 million; International Development Association, $218.6 million) together provided 56.9% of total DAAH across the study period (eFigure 3 in the Supplement). Sexual and reproductive health and HIV and AIDS have been the major focuses of all top 5 donors; for example, the Global Fund to Fight AIDS, Tuberculosis and Malaria donated $748.7 million to these 2 areas, accounting for 92.8% of its total DAAH disbursement. When using adolescent-inclusive estimates, the United States was the largest donor (eFigure 4 in the Supplement). Similarly, most of the investment made by the United States went to HIV and AIDS prevention, diagnosis, and treatment, as well as to reproductive health.

### Estimates of Per-Adolescent Aid Disbursed to Adolescent-Targeted DAAH by Recipient Region and Recipient Country

Table 2 presents trends of DAAH per adolescent by recipient region and income between 2003 and 2015. The annual DAAH per adolescent in a region and income group is calculated as the total DAAH for all included countries in that region and income group divided by the total adolescent population of those countries in a year. Overall, DAAH per adolescent increased steadily over time by 4-fold, from $0.06 in 2003 to $0.24 in 2015. The annual growth rate reached 12.3%. Among the 50 countries in Africa, 49 of them belong to sub-Saharan Africa, except for Algeria. The cumulative DAAH per adolescent in sub-Saharan Africa was the highest among all regions ($5.37). Except for Europe and Southeast Asia, LICs have received higher cumulative DAAH per adolescent than lower-middle–income countries (LMCs) and upper-middle–income countries in each region. The Americas (North America, Central America, and South America) were the only regions with LMCs receiving more DAAH per adolescent than LICs for most years, but Haiti was the only LIC in the Americas, and, in 1 year (2009), its DAAH per capita reached as high as $8.93, with major efforts to control HIV and AIDS and improve reproductive health. The Western Pacific region received the least amount of DAAH per adolescent in almost all the years studied. However, its LICs received $1.00 DAAH per adolescent in 2015, the second-highest amount among all regions and income groups in that year. The adolescent-inclusive estimates show similar results (eTable 17 in the Supplement).

**Table 2.** Adolescent-Targeted DAAH per Adolescent by Recipient Region and Income Classification, 2003-2015^a^

Characteristic	Adolescent-Targeted DAAH per Adolescent, US Dollars													Annual Growth Rate, %^b^
	2003	2004	2005	2006	2007	2008	2009	2010	2011	2012	2013	2014	2015	
Overall^c^	0.06	0.08	0.09	0.09	0.10	0.09	0.15	0.17	0.17	0.20	0.21	0.24	0.24	12.25
LICs	0.19	0.21	0.20	0.23	0.35	0.29	0.52	0.44	0.44	0.69	0.67	0.82	0.89	13.64
LMCs	0.05	0.09	0.11	0.09	0.08	0.07	0.10	0.16	0.15	0.15	0.15	0.16	0.12	7.38
UMCs	0.03	0.02	0.03	0.04	0.04	0.05	0.06	0.07	0.08	0.05	0.07	0.07	0.08	9.23
Recipient region^c^														
Africa	0.19	0.24	0.22	0.19	0.23	0.20	0.37	0.44	0.41	0.61	0.58	0.69	0.80	12.73
SSA	0.21	0.26	0.23	0.20	0.24	0.21	0.39	0.45	0.42	0.63	0.60	0.71	0.82	12.22
LICs	0.27	0.30	0.26	0.28	0.36	0.28	0.52	0.58	0.56	0.95	0.80	0.92	1.18	13.12
LMCs	0.11	0.21	0.21	0.10	0.09	0.11	0.16	0.31	0.25	0.20	0.34	0.41	0.29	8.22
UMCs	0.12	0.05	0.03	0.05	0.03	0.10	0.34	0.07	0.12	0.13	0.12	0.29	0.26	6.67
Americas	0.09	0.06	0.12	0.17	0.21	0.15	0.34	0.24	0.24	0.21	0.29	0.26	0.26	9.24
LICs	0.09	0.04	0.37	1.52	3.44	0.13	8.93	0.57	0.68	0.25	0.38	0.54	0.29	9.94
LMCs	0.45	0.34	0.60	0.83	0.74	0.77	0.98	1.40	1.50	1.37	1.60	1.52	0.99	6.69
UMCs	0.06	0.03	0.06	0.06	0.08	0.08	0.07	0.10	0.08	0.07	0.14	0.10	0.17	9.84
Eastern Mediterranean	0.07	0.11	0.03	0.07	0.05	0.05	0.12	0.16	0.16	0.18	0.13	0.13	0.17	7.67
LICs	0.00	0.02	0.17	0.01	0.03	0.01	0.16	0.36	0.96	0.23	0.21	0.58	0.86	39.52
LMCs	0.08	0.11	0.02	0.07	0.05	0.06	0.12	0.15	0.11	0.20	0.14	0.09	0.11	2.66
UMCs	0.07	0.23	0.03	0.06	0.07	0.06	0.10	0.10	0.10	0.08	0.07	0.13	0.14	5.98
Europe	0.11	0.17	0.15	0.26	0.37	0.33	0.30	0.32	0.35	0.24	0.28	0.25	0.23	6.34
LICs	0.24	0.32	0.36	0.08	0.09	0.18	0.03	0.23	0.21	0.30	0.49	0.58	0.37	3.49
LMCs	0.13	0.20	0.10	0.21	0.53	0.36	0.46	0.49	0.37	0.32	0.21	0.28	0.31	7.27
UMCs	0.08	0.14	0.17	0.31	0.30	0.33	0.22	0.22	0.35	0.17	0.29	0.19	0.17	6.32
Southeast Asia	0.02	0.05	0.09	0.06	0.06	0.07	0.06	0.10	0.11	0.07	0.10	0.15	0.05	7.93
LICs	0.04	0.07	0.08	0.11	0.22	0.36	0.22	0.14	0.07	0.16	0.45	0.63	0.22	14.15
LMCs	0.01	0.04	0.10	0.06	0.03	0.03	0.02	0.06	0.08	0.06	0.05	0.06	0.02	6.35
UMCs	0.02	0.02	0.03	0.04	0.01	0.01	0.33	1.05	1.02	0.14	0.06	0.13	0.10	12.12
Western Pacific	0.01	0.01	0.02	0.01	0.02	0.01	0.02	0.03	0.03	0.05	0.04	0.02	0.04	12.25
LICs	0.24	0.05	0.06	0.16	0.47	0.36	0.37	0.28	0.76	0.75	0.27	0.66	1.00	12.73
LMCs	0.04	0.09	0.11	0.06	0.06	0.05	0.09	0.13	0.09	0.19	0.21	0.08	0.14	10.38
UMCs	0.00	0.00	0.00	0.00	0.00	0.00	0.01	0.00	0.00	0.01	0.00	0.00	0.00	0.14

Abbreviations: DAAH, development assistance for adolescent health; LICs, low-income countries; LMCs, lower-middle–income countries; SSA, sub-Saharan Africa; UMCs, upper-middle–income countries.

^a^ Unallocable or regional DAAH projects were not included. All disbursement data were converted to constant 2015 US dollars. We calculated DAAH per adolescent for each category with the formula: total DAAH disbursements to all countries in that category/total population of all countries in that category. For example, DAAH per adolescent in Africa equals the sum of DAAH to each African country divided by the sum of adolescent population in each African country. If a country was missing data on adolescent population or DAAH disbursement, we excluded the country from our analysis.

^b^ Annual growth rate = (end value/start value)^1/period^ − 1.

^c^ Based on 2010 World Bank income classification.

In terms of cumulative DAAH per adolescent at the country level (eTable 18 in the Supplement), countries with small adolescent populations, such as Vanuatu ($111.6, the highest), Guyana ($85.9, the second highest), Cabo Verde ($82.7, the third highest), Kiribati ($69.7, the fourth highest), and Belize ($66.1, the fifth highest), received the highest cumulative DAAH per adolescent. Of countries ranked in the top 10 of cumulative DAAH per adolescent between 2003 and 2015, two were LICs (Sierra Leone and Malawi), 6 were LMCs (Vanuatu, Guyana, Cabo Verde, Kiribati, Belize, and São Tomé and Principe), and 2 were upper-middle–income countries (Grenada and Bosnia and Herzegovina). Most of the top 10 countries belong to the middle-income group with small populations. Adolescent-inclusive estimates showed a similar trend (eTable 19 in the Supplement).

**Figure 2.**
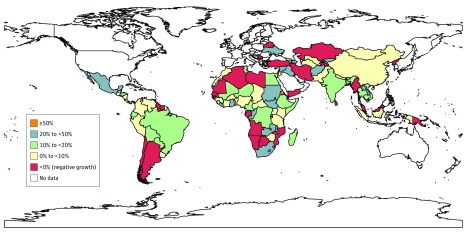
Annual Growth Rate in Adolescent-Targeted Development Assistance for Adolescent Health per Adolescent, 2003-2015 Unallocable or regional development assistance for adolescent health projects were not included. All disbursement data were converted to constant 2015 US dollars. Annual growth rate = (end value/start value)^1/period^ − 1. For example, development assistance for adolescent health per adolescent in Bangladesh was $0.035 in 2003 (start value) and $0.312 in 2015 (end value). The period is 2015 − 2003 = 12 years. The compound annual growth rate for Bangladesh is (0.312/0.035)^1/12^ − 1 = 19.94%.

Figure 2 summarizes the annual growth rates of DAAH per adolescent at the country level. We established 5 groups based on the compound annual growth rate. Three countries had growth rates of 50% or more, including 1 LIC (Eritrea) and 2 LMCs (São Tomé and Principe and Tonga). São Tomé and Principe had the highest annual growth rate (149.4%), largely owing to the extremely low DAAH per adolescent in the early years of coverage. A total of 26 countries had annual growth rates of DAAH per adolescent above 20% and below 50%, including 20 LICs or LMCs (eg, Burundi, Malawi, and Zambia). A total of 28 countries had moderate annual growth rates above 10% and below 20%, including 22 LICs or LMCs (eg, Cambodia, Ethiopia, and Mali). The DAAH per adolescent in another 39 countries increased above 0% but was below 10%, including 28 LICs or LMCs (eg, Uganda, Chad, and Benin). There were 34 countries with negative annual growth rates, including 5 LICs (eg, Gambia, Mozambique, and Tajikistan), 9 LMCs (eg, Yemen, Kiribati, and Senegal), and 20 upper-middle–income countries (eg, Turkey, Chile, and Algeria) (eTable 18 in the Supplement). The annual growth rates of adolescent-inclusive DAAH are presented in eFigure 5 in the Supplement.

### Adolescent-Targeted DAAH Disbursements Targeting the Leading DALYs of Adolescents

Table 3 presents the top 10 DALYs for the 132 countries involved in our study in 2015 and the corresponding DAAH they received targeting these DALYs. In the overall setting, skin and subcutaneous diseases accounted for the largest amount of DALYs (7.6% of total DALYs for adolescents [17 561 626.05 of 231 030 476.65]) and were the leading cause of DALYs in 2015. Overall, HIV and AIDS received the largest amount of adolescent-targeted DAAH in all settings, followed by interpersonal violence, tuberculosis, and diarrheal diseases. Other leading causes of DALYs, such as skin and subcutaneous diseases, low back and neck pain, road injury, iron-deficiency anemia, and depressive disorders, received either no DAAH or a very small amount of DAAH. The results presented by adolescent-inclusive estimates are similar.

**Table 3.** Top 10 Causes of DALYs for the 132 Countries and the Corresponding DAAH to the DALYs in 2015

Analysis Level for Top 10 Causes^a^	DALYs (% of Total DALYs)	DAAH, in Millions of US$ (% of Total DAAH)	
		Adolescent Targeted	Adolescent Inclusive
Low-income countries			
HIV and AIDS	4 673 589 (8.8)	126.4 (49.1)	239.4 (8.3)
Skin and subcutaneous diseases	3 386 513 (6.4)	0	0
Road injuries	2 704 170 (5.1)	0	10.0 (0.3)
Diarrheal diseases	2 202 433 (4.1)	2.5 (1.0)	136.6 (4.7)
Tuberculosis	1 881 594 (3.5)	1.4 (0.6)	197.0 (6.8)
Iron-deficiency anemia	1 479 313 (2.8)	1.2 (0.5)	4.6 (0.2)
Depressive disorders	1 423 021 (2.7)	0	4.9 (0.2)
Interpersonal violence	1 183 114 (2.2)	10.6 (4.1)	42.2 (1.5)
Low back and neck pain	1 084 142 (2.0)	0	0
Self-harm	880 951 (1.7)	0.1 (0.0)	75.0 (2.6)
Lower-middle–income countries			
Skin and subcutaneous diseases	8 393 250 (7.0)	0	0
Road injuries	7 352 028 (6.1)	1.3 (1.5)	7.8 (0.3)
Iron-deficiency anemia	6 362 196 (5.3)	0.6 (0.7)	3.3 (0.1)
Self-harm	5 469 983 (4.5)	0	31.4 (1.4)
Diarrheal diseases	3 297 245 (2.7)	2.5 (2.9)	80.0 (3.5)
HIV and AIDS	3 208 219 (2.7)	9.9 (11.2)	48.9 (2.2)
Depressive disorders	3 125 132 (2.6)	0	6.1 (0.3)
Tuberculosis	2 773 472 (2.3)	0.1 (0.1)	153.2 (6.8)
Low back and neck pain	2 741 997 (2.3)	0	0
Interpersonal violence	2 154 684 (1.8)	13.1 (14.7)	51.5 (2.3)
Upper-middle–income countries			
Skin and subcutaneous diseases	5 781 863 (10.1)	0	0.1
Road injuries	5 573 694 (9.7)	0.1 (0.2)	0.6 (0.1)
Interpersonal violence	3 432 025 (6.0)	4.7 (11.0)	26.9 (3.4)
Low back and neck pain	2 190 603 (3.8)	0	0
Depressive disorders	2 159 973 (3.8)	3.3 (7.9)	4.3 (0.5)
Self-harm	1 729 790 (3.0)	2.9 (6.8)	17.7 (2.2)
HIV and AIDS	1 267 458 (2.2)	20.4 (48.1)	68.1 (8.6)
Iron-deficiency anemia	676 405 (1.2)	0	0.1 (0.0)
Diarrheal diseases	424 985 (0.7)	0.05 (0.1)	5.5 (0.7)
Tuberculosis	382 638 (0.7)	7.7 (18.1)	84.6 (10.6)
Overall			
Skin and subcutaneous diseases	17 561 626 (7.6)	0	0.1 (0.0)
Road injuries	15 629 892 (6.8)	1.4 (0.3)	18.4 (0.2)
HIV and AIDS	9 149 265 (4.0)	156.7 (29.7)	356.4 (4.4)
Iron-deficiency anemia	8 517 914 (3.7)	1.9 (0.4)	7.9 (0.1)
Self-harm	8 080 725 (3.5)	3.0 (0.6)	124.1 (1.5)
Interpersonal violence	6 769 823 (2.9)	28.4 (5.4)	120.7 (1.5)
Depressive disorders	6 708 125 (2.9)	3.4 (0.6)	15.3 (0.2)
Low back and neck pain	6 016 741 (2.6)	0	0
Diarrheal diseases	5 924 662 (2.6)	5.1 (1.0)	222.1 (2.8)
Tuberculosis	5 037 705 (2.2)	9.2 (1.7)	434.8 (5.4)

Abbreviations: DAAH, development assistance for adolescent health; DALYs, disability-adjusted life-years.

^a^ Based on 2010 World Bank income classification.

## Discussion

Using data from the CRS, we tracked DAAH disbursements to 132 recipient countries. We found that, despite the volume of adolescent-targeted DAAH increasing 5-fold between 2003 and 2015, it accounted for only a small share of total DAH (2.2% in 2015). Considering that one-fourth of the world’s population are adolescents (10-24 years of age), it is clear that adolescent health has not been a priority of donors.^18^ The Global Fund to Fight AIDS, Tuberculosis and Malaria; the United Nations Population Fund; the United States; the United Kingdom; and the International Development Association were the top 5 leading donors of adolescent-targeted DAAH, and together they contributed $2.1 billion DAAH (56.9% of total adolescent-targeted DAAH). Between 2014 and 2015, the United Kingdom was the driving force for the increase in DAAH, and its disbursements to DAAH increased by $77.5 million. Consequently, the total DAAH increased by $67.3 million, from $461.2 million in 2014 to $528.5 million in 2015. Sexual and reproductive health and HIV and AIDS have been the major focus of DAAH, which received 68.0% of total adolescent-targeted DAAH between 2003 and 2015.

Among all regions, Africa received the highest cumulative DAAH per adolescent, and the Western Pacific received the least. However, the DAAH per adolescent of LICs in the Western Pacific is close to that in Africa ($1.00 vs $1.18 in 2015). At the country level, countries that received the highest amount of DAAH per adolescent were mostly less populous, with small populations of adolescents. This finding was consistent with previous studies showing a negative association between development assistance per capita and population size.^30,31,32^

Among the top 10 leading DALYs in 2015, HIV and AIDS had the largest amount of DAAH per adolescent in all income groups. Other leading causes of DALYs, such as road injuries, depressive disorder, and iron-deficiency anemia, received either no DAAH or very small amounts of DAAH, suggesting that the allocation of DAAH does not match well with the burden of diseases. Our results align with those of Skirbekk et al^33^ that demonstrate a mismatch between causes of disease burden and allocation of DAH.

### Limitations

There are some limitations in these analyses. First, the key word searching strategy was unable to capture all DAAH projects; our validation study determined that roughly 0.15% of DAAH projects were not captured. Second, the quality of project descriptions from donors is a further potential limitation, even though there was no evidence to show that the current reporting system would introduce systematic bias in the estimates of DAAH relative to DAH. Third, the adolescent-targeted estimates excluded projects in which adolescents were a partial target and may have underestimated DAAH. In contrast, the adolescent-inclusive estimates included projects with purposes unrelated to adolescent health and may have overestimated DAAH. Fourth, we used the proportion of adolescents in the total population or population of reproductive age to allocate projects that could benefit adolescents but that did not explicitly refer to adolescents. We found that, although adolescents (10-24 years of age) accounted for one-fourth of the world’s population in 2015, their DALYs accounted for 11% of the total DALYs.^18,29^ Our allocation method may therefore lead to an overestimation of adolescent-inclusive DAAH. Further limits include the missingness in CRS disbursements before 2007 (eg, missingness rates in the health sector between 2003 and 2006 ranging from 7% to 16%)^16,20^ and the unavailability of aid data from new donors (eg, China) and private foundations apart from the Bill & Melinda Gates Foundation.

## Conclusions

This study suggests a need to reconsider patterns and levels of international investment in adolescent health. Although DALYs of adolescents accounted for 11% of global DALYs in 2015, the aid disbursed to adolescent-targeted DAAH was only 2.2% of total DAH. This finding suggests that levels of DAAH fall well short of that disbursed to projects targeted at other age groups. This inequity is perhaps not surprising given that adolescent health has not been a priority during the era of the United Nations Millennium Development Goals. However, with the introduction of the Global Strategy for Women’s, Children’s and Adolescents’ Health, much larger investments in adolescent health are likely to be needed. The need for investment in adolescent health in LICs and middle income countries is further heightened by the very large generation of adolescents currently growing up in these countries.

The recent major focal areas for DAAH have been on sexual and reproductive health and HIV and AIDS, with other leading treatable or preventable causes of disease burden, including anemia, mental disorders, or road traffic injuries, largely overlooked. Interventions targeting several of these areas have been found to have high benefit to cost ratios, even without considering the intergenerational benefits.^2^ For example, the benefit to cost ratio for mental health care was recently estimated to be 5.3, and the cost to benefit ratio for injury prevention was recently estimated to be 5.9.^2^ Although investments in sexual and reproductive health and HIV and AIDS should undoubtedly be maintained and arguably extended, donors should consider investments in other causes of health risk and disease burden with effective interventions and a strong economic case. Last, there is a need for greater investment in evaluations of the effect of action in areas where cost-effectiveness data are still limited, particularly in a local regional context. Such evidence would be invaluable in guiding future resource allocations for adolescent health in comparison with other areas of need.

## Notes

Supplement. **eTable 1.** List of Donor Countries Included in the CRS **eTable 2.** 132 Recipients in the CRS (According to the World Health Organization Regions) **eTable 3.** Key Words to Identify the Related Age Group (Adolescence) in the Creditor Reporting System **eTable 4.** CRS Purpose Name and Respective Fractions Allocated to Adolescent Health **eTable 5.** Definitions of DAAH on the Leading Causes of DALYs of Adoelscent Health **eTable 6.** Key Words Used to Search for Projects on Skin and Subcutaneous Diseases in the Creditor Reporting System **eTable 7.** Key Words Used to Search for Road Injury Projects in the Creditor Reporting System **eTable 8.** Key Words Used to Search for HIV/AIDS Projects in the Creditor Reporting System **eTable 9.** Key Words Used to Search for Projects on Iron-Deficiency Anemia in the Creditor Reporting System **eTable 10.** Key Words Used to Search for Self-Harm Projects in the Creditor Reporting System **eTable 11.** Key Words Used to Search for Projects on Interpersonal Violence in the Creditor Reporting System **eTable 12.** Key Words Used to Search for Projects on Depressive Disorders in the Creditor Reporting System **eTable 13.** Key Words Used to Search for Projects on Lower Back and Neck Pain in the Creditor Reporting System **eTable 14.** Key Words Used to Search for Diarrheal Projects in the Creditor Reporting System **eTable 15.** Key Words Used to Search for Tuberculosis Projects in the Creditor Reporting System **eTable 16.** Annual DAAH (Adolescent Targeted) and DAH Disbursement ($ million), 2003-2015 **eTable 17.** DAAH (Adolescent Inclusive) per Adolescent (US$) by Recipient Region and Income Classification, 2003-2015 **eTable 18.** DAAH (Adolescent Targeted) per Adolescent (US$) by Recipient Country, 2003-2015 **eTable 19.** DAAH (Adolescent Inclusive) per Adolescent (US$) by Recipient Country, 2003-2015 **eFigure 1.** The Proportion of DAAH (Adolescent Targeted) in DAH (%), 2003-2015 **eFigure 2.** Trends in Annual DAAH (Adolescent Inclusive) and Annual SRH or HIV/AIDS Disbursement, 2003-2015 (million US$) **eFigure 3.** Annual DAAH (Adolescent Targeted) by Donors, 2003-2015 (million US$) **eFigure 4.** Annual DAAH (Adolescent Inclusive) by Donors, 2003-2015 (million US$) **eFigure 5.** Annual Growth Rate in DAAH (Adolescent Inclusive) per Adolescent, 2003-2015 (in percentage) **eReferences**
