# Supplementary material for: Global Development Assistance for Adolescent Health From 2003 to 2015
Source: JAMA Netw Open. 2018 Aug 10;1(4):e181072. doi: 10.1001/jamanetworkopen.2018.1072 (PMC6324521; doi:10.1001/jamanetworkopen.2018.1072)
Supplement: Supplement. — eTable 1. List of Donor Countries Included in the CRS eTable 2. 132 Recipients in the CRS (According to the World Health Organization Regions) eTable 3. Key Words to Identify the Related Age Group (Adolescence) in the Creditor Reporting System eTable 4. CRS Purpose Name and Respective Fractions Allocated to Adolescent Health eTable 5. Definitions of DAAH on the Leading Causes of DALYs of Adoelscent Health eTable 6. Key Words Used to Search for Projects on Skin and Subcutaneous Diseases in the Creditor Reporting System eTable 7. Key Words Used to Search for Road Injury Projects in the Creditor Reporting System eTable 8. Key Words Used to Search for HIV/AIDS Projects in the Creditor Reporting System eTable 9. Key Words Used to Search for Projects on Iron-Deficiency Anemia in the Creditor Reporting System eTable 10. Key Words Used to Search for Self-Harm Projects in the Creditor Reporting System eTable 11. Key Words Used to Search for Projects on Interpersonal Violence in the Creditor Reporting System eTable 12. Key Words Used to Search for Projects on Depressive Disorders in the Creditor Reporting System eTable 13. Key Words Used to Search for Projects on Lower Back and Neck Pain in the Creditor Reporting System eTable 14. Key Words Used to Search for Diarrheal Projects in the Creditor Reporting System eTable 15. Key Words Used to Search for Tuberculosis Projects in the Creditor Reporting System eTable 16. Annual DAAH (Adolescent Targeted) and DAH Disbursement ($ million), 2003-2015 eTable 17. DAAH (Adolescent Inclusive) per Adolescent (US$) by Recipient Region and Income Classification, 2003-2015 eTable 18. DAAH (Adolescent Targeted) per Adolescent (US$) by Recipient Country, 2003-2015 eTable 19. DAAH (Adolescent Inclusive) per Adolescent (US$) by Recipient Country, 2003-2015 eFigure 1. The Proportion of DAAH (Adolescent Targeted) in DAH (%), 2003-2015 eFigure 2. Trends in Annual DAAH (Adolescent Inclusive) and Annual SRH or HIV/AIDS Disbursement, 2003-2015 (million [file jamanetwopen-1-e181072-s001.pdf]

Supplementary Online Content

Li Z, Li M, Patton GC, Lu C. Global development assistance for adolescent health from 2003 to 2015. *JAMA Netw Open.* 2018;1(4):e181072. doi:10.1001/jamanetworkopen.2018.1072

eTable 1. List of Donor Countries Included in the CRS

eTable 2. 132 Recipients in the CRS (According to the World Health Organization Regions)

eTable 3. Key Words to Identify the Related Age Group (Adolescence) in the Creditor Reporting System

eTable 4. CRS Purpose Name and Respective Fractions Allocated to Adolescent Health

eTable 5. Definitions of DAAH on the Leading Causes of DALYs of Adolescent Health

eTable 6. Key Words Used to Search for Projects on Skin and Subcutaneous Diseases in the Creditor Reporting System

eTable 7. Key Words Used to Search for Road Injury Projects in the Creditor Reporting System

eTable 8. Key Words Used to Search for HIV/AIDS Projects in the Creditor Reporting System

eTable 9. Key Words Used to Search for Projects on Iron-Deficiency Anemia in the Creditor Reporting System

eTable 10. Key Words Used to Search for Self-Harm Projects in the Creditor Reporting System

eTable 11. Key Words Used to Search for Projects on Interpersonal Violence in the Creditor Reporting System

eTable 12. Key Words Used to Search for Projects on Depressive Disorders in the Creditor Reporting System

eTable 13. Key Words Used to Search for Projects on Lower Back and Neck Pain in the Creditor Reporting System

eTable 14. Key Words Used to Search for Diarrheal Projects in the Creditor Reporting System

eTable 15. Key Words Used to Search for Tuberculosis Projects in the Creditor Reporting System

eTable 16. Annual DAAH (Adolescent Targeted) and DAH Disbursement ($ million), 2003-2015

eTable 17. DAAH (Adolescent Inclusive) per Adolescent (US$) by Recipient Region and Income Classification, 2003-2015

eTable 18. DAAH (Adolescent Targeted) per Adolescent (US$) by Recipient Country, 2003-2015

eTable 19. DAAH (Adolescent Inclusive) per Adolescent (US$) by Recipient Country, 2003-2015

eFigure 1. The Proportion of DAAH (Adolescent Targeted) in DAH (%), 2003-2015

eFigure 2. Trends in Annual DAAH (Adolescent Inclusive) and Annual SRH or HIV/AIDS Disbursement, 2003-2015 (million US$)

eFigure 3. Annual DAAH (Adolescent Targeted) by Donors, 2003-2015 (million US$)

eFigure 4. Annual DAAH (Adolescent Inclusive) by Donors, 2003-2015 (million US$)

eFigure 5. Annual Growth Rate in DAAH (Adolescent Inclusive) per Adolescent, 2003-2015 (in percentage)

eReferences

This supplementary material has been provided by the authors to give readers additional information about their work.

© 2018 Li Z et al. *JAMA Network Open.*

eTable 1. List of Donor Countries Included in the CRS

DAC members		Non-DAC multilateral donors		Non-DAC donors		Private donors	
Australia	2003-2015	Adaptation fund	2010-2015	Azerbaijan	2014-2015	Bill & Melinda Gates Foundation	2009-2015
Austria	2003-2015	African Development Bank	2003-2015	Bulgaria	2015		
Belgium	2003-2015	African Development Fund	2003-2015	Chinese Taipei	2015		
Canada	2003-2015	Arab Bank for Economic Development in Africa	2003-2015	Croatia	2014-2015		
Czech Republic	2011-2015	Arab Fund (AFESD)	2008-2015	Cyprus	2014-2015		
Denmark	2003-2015	AsDB Special Funds	2003-2015	Estonia	2013-2015		
EU Institutions	2003-2015	Asian Development Bank(AsDB)	2003-2015	Israel	2015		
Finland	2003-2015	Council of Europe Development Bank	2010-2015	Kazakhstan	2013-2015		
France	2003-2015	Food and Agriculture Organization	2013	Kuwait (KFAED)	2010-2015		
Germany	2003-2015	Global Alliance for Vaccines and Immunisation	2007-2015	Latvia	2015		
Greece	2003-2015	Global Environment Facility	2003-2015	Liechtenstein	2015		
Hungary	2014-2015	Global Fund to Fight AIDS, Tuberculosis and Malaria	2003-2015	Lithuania	2014-2015		
Iceland	2011-2015	Global Green Growth Institute	2013-2015	Malta	2015		
Ireland	2003-2015	Inter-American Development Bank Special Fund	2003-2015	Romania	2014-2015		
Italy	2003-2015	International Development Association	2003-2015	Russia	2015		
Japan	2003-2015	International Fund for Agricultural Development	2003-2015	Saudi Arabia	2015		
Korea	2003, 2006-	International Labour Organization	2012-	Thailand	2015		

© 2018 Li Z et al. *JAMA Network Open.*

DAC members		Non-DAC multilateral donors		Non-DAC donors		Private donors	
	2015		2015				
Luxembourg	2003-2015	International Monetary Fund	2003-2015	Timor-Leste	2014-2015		
Netherlands	2003-2015	International Monetary Fund	2003-2015	Turkey	2015		

© 2018 Li Z et al. *JAMA Network Open.*

DAC members		Non-DAC multilateral donors		Non-DAC donors		Private donors	
New Zealand	2003-2015	Islamic Development Bank	2003-2015	United Arab Emirates	2009-2015		
Norway	2003-2015	Joint United Nations Programme on HIV/AIDS	2003-2015				
Poland	2013-2015	Nordic Development Fund	2009-2015				
Portugal	2003-2015	OPEC Fund for International Development	2009-2015				
Slovak Republic	2013-2015	Organization for Security and Co-operation in Europe	2010-2015				
Slovenia	2010-2015	United Nations Children's Fund	2003-2015				
Spain	2003-2015	United Nations Development Programme	2004-2015				
Sweden	2003-2015	United Nations Economic Commission for Europe	2008-2015				
Switzerland	2003-2015	United Nations Peacebuilding Fund	2007-2015				
United Kingdom	2003-2015	United Nations Population Fund	2003-2015				
United States	2003-2015	United Nations Refugee Agency	2011-2015				
		World Food Programme	2008-2015				
		World Health Organization	2009-2015				

© 2018 Li Z et al. *JAMA Network Open.*

eTable 2. 132 Recipients in the CRS (According to the World Health Organization Regions)

African countries (50)<br>*SSA:* Angola, Benin, Botswana, Burkina Faso, Burundi, Cabo Verde, Cameroon, Central African Republic, Chad, Comoros, Congo, Côte d'Ivoire, Democratic Republic of the Congo, Djibouti, Equatorial Guinea, Eritrea, Ethiopia, Gabon, Gambia, Ghana, Guinea, Guinea-Bissau, Kenya, Lesotho, Liberia, Madagascar, Malawi, Mali, Mauritania, Mauritius, Mozambique, Namibia, Niger, Nigeria, Rwanda, Sao Tome and Principe, Senegal, Seychelles, Sierra Leone, Somalia, South Africa, South Sudan, Sudan, Swaziland, Togo, Uganda, United Republic of Tanzania, Zambia, Zimbabwe<br>*LICs:* Benin, Burkina Faso, Burundi, Central African Republic, Chad, Comoros, Democratic Republic of the Congo, Eritrea, Ethiopia, Gambia, Guinea, Guinea-Bissau, Kenya, Liberia, Madagascar, Malawi, Mali, Mozambique, Niger, Rwanda, Sierra Leone, Somalia, South Sudan, Togo, Uganda, Tanzania, Zimbabwe<br>*LMCs*: Angola, Cabo Verde, Cameroon, Congo, Côte d'Ivoire, Djibouti, Ghana, Lesotho, Mauritania, Nigeria, Sao Tome and Principe, Senegal, Sudan, Swaziland, Zambia<br>*UMCs*: Algeria, Botswana, Equatorial Guinea, Gabon, Mauritius, Namibia, Seychelles, South Africa
American countries (26):<br>*LICs:* Haiti<br>*LMCs*: Belize, Bolivia, El Salvador, Guatemala, Guyana, Honduras, Nicaragua, Paraguay<br>*UMCs:* Argentina, Brazil, Chile, Colombia, Costa Rica, Cuba, Dominican Republic, Ecuador, Grenada, Jamaica, Mexico, Panama, Peru, Saint Vincent and the Grenadines, Suriname, Uruguay, Venezuela (Bolivarian Republic of)
Eastern Mediterranean (12):<br>*LICs:* Afghanistan<br>*LMCs*: Egypt, Iraq, Morocco, Pakistan, Syrian Arab Republic, Yemen<br>*UMCs:* Iran, Jordan, Lebanon, Libya, Tunisia
Europe (18):<br>*LICs:* Kyrgyzstan, Tajikistan<br>*LMCs*: Armenia, Georgia, Moldova, Turkmenistan, Ukraine, Uzbekistan<br>*UMCs:* Mayotte, Albania, Azerbaijan, Belarus, Bosnia and Herzegovina, Kazakhstan, Montenegro, Serbia, The former Yugoslav Republic of Macedonia, Turkey
South-East Asia (11):<br>*LICs:* Bangladesh, Democratic People's Republic of Korea, Myanmar, Nepal<br>*LMCs*: Bhutan, India, Indonesia, Sri Lanka, Timor-Leste, Maldives, Thailand<br>*UMCs:* Maldives, Thailand
Western Pacific (15):<br>*LICs:* Cambodia<br>*LMCs*: Fiji, Kiribati, Lao People's Democratic Republic, Micronesia (Federated States of), Mongolia, Papua New Guinea, Philippines, Samoa, Solomon Islands, Tonga, Vanuatu, Viet Nam<br>*UMCs:* China, Malaysia
15 states were excluded: Anguilla, Croatia, Cook Islands, Kosovo, Marshall Islands, Montserrat, Nauru, Niue, Palau, Saint Helena, Saint Kitts and Nevis, Tokelau, Tuvalu, Wallis and Futuna, West Bank and Gaza Strip

Note: "SSA" represents "Sub-Saharan Africa"; "LICs" represents "Low-income countries"; "LMCs" represents "Lower-middle income countries"; "UMCs" represents "Upper-middle income countries".

© 2018 Li Z et al. *JAMA Network Open.*

eTable 3. Key Words to Identify the Related Age Group (Adolescence) in the Creditor Reporting System

Adolescent	adolescence	adolescente	between 10 and 14
between 10 and 15	between 10 and 19	between 10 and 20	between 10 and 24
between 15 and 19	between 15 and 20	between 15 and 24	between 19 and 24
between 20 and 24	excluded young	excluded youth	fifteen to nineteen
fifteen to twenty	fifteen to twenty-four	freshman	high school
middle school	nineteen to twenty-dour	older boy	older girl
out of school young	out of school youth	out-of-school young	out-of-school youth
over-age school student	overage school student	post-secondary school	secondary-school
sophomore	teen	teenager	ten to fifteen
ten to fourteen	ten to nineteen	ten to twenty	ten to twenty-four
twenty to twenty-four	vocational school	young	young men
young mother	young women	youth	yorth
10-14	10-15	10-19	10-20
10-24	15-19	15-20	15-24
19-24	20-24	10-14 years old	10-15 years old
10-19 years old	10-20 years old	10-24 years old	15-19 years old
15-20 years old	15-24 years old	19-24 years old	20-24 years old
10 to 14	10 to 15	10 to 19	10 to 20
10 to 24	15 to 19	15 to 20	15 to 24
19 to 24	20 to 24		

Note:

1. We have translated all the keywords listed above to Spanish, French, Portuguese, Italian, Dutch, German, Norwegian, and Swedish
2. We performed keywords searching using 1) all lower case; 2) all upper case; 3) capitalize the first letter of each word 4) capitalize the first letter of the term
3. To prevent missing keywords containing unrecognizable characters (e.g. “t?eenager”), we replaced each letter of the keywords in the table with “?”, “??” “/” or spacing.

© 2018 Li Z et al. *JAMA Network Open.*

eTable 4. CRS Purpose Name and Respective Fractions Allocated to Adolescent Health

CRS purpose	Fraction of funds on adolescent health	Source of the fraction
General budget support	Country value for each year	1. The proportion of government spending that goes to health, from National Health Accounts database<br>2. The proportion of adolescents/total population in developing countries, from UNDP
Basic health infrastructure	Country value for each year	Country-specific proportion of adolescents/total population
Family planning	Country value for each year	Country-specific proportion of adolescents (15-24 years)/population aged 15-49 years , UNDP
Health education	Country value for each year	Country-specific proportion of adolescents/total population
Health personnel development	Country value for each year	Country-specific proportion of adolescents/total population
Infectious disease control	Country value for each year	Country-specific proportion of adolescents/total population
Malaria control	Country value for each year	Country-specific proportion of adolescents/total population
Medical education/training	Country value for each year	Country-specific proportion of adolescents/total population
Medical research	Country value for each year	Country-specific proportion of adolescents/total population
Medical services	Country value for each year	Country-specific proportion of adolescents/total population
Personnel development for population & reproductive health	Country value for each year	Country-specific proportion of adolescents/total population
Reproductive health care	Country value for each year	Country-specific proportion of adolescents (15-24 years)/the total population aged 15-49 years, UNDP
STD control including HIV/AIDS	Country value for each year	Country-specific proportion of adolescents/total population
Tuberculosis control	Country value for each year	Country-specific proportion of adolescents/total population
Road transport	Country value for each year	Country-specific proportion of adolescents/total population

© 2018 Li Z et al. *JAMA Network Open.*

CRS purpose	Fraction of funds on adolescent health	Source of the fraction
Basic drinking water supply	Country value for each year	Country-specific proportion of adolescents/total population
Basic drinking water supply and basic sanitation	Country value for each year	Country-specific proportion of adolescents/total population
Basic sanitation	Country value for each year	Country-specific proportion of adolescents/total population

© 2018 Li Z et al. *JAMA Network Open.*

eTable 5. Definitions of DAAH on the Leading Causes of DALYs of Adolescent Health

**DALYs/Mortality causes/Risk factors**	**Total DALYs between 2003 and 2015 in the 132 studied countries**	**Definition**
Skin and subcutaneous diseases	17,561,626	Funding to projects that specified their activities to prevent, diagnose, or treat skin and subcutaneous diseases, such as dermatitis, psoriasis, cellulitis, pyoderma, scabies, etc.(1,2)
Road Injuries	15,629,892	Funding to projects that specified their activities to reduce fatal or non-fatal injuries incurred as a result of a road traffic crash among adolescents, which occurs on a public road and involving at least one moving vehicle.(3)
HIV/AIDS	9,149,265	Funding to projects that specified their activities as providing adolescents with HIV/AIDS prevention, diagnosis, counselling, treatment, psychosocial support, family planning, and promotion of contraception.(4–6)
Iron-deficiency anaemia	8,517,914	Funding to projects that specified their activities to prevent, diagnose, or treat the condition in which blood lacks adequate healthy red blood cells due to insufficient iron. (7,8)
Self-harm	8,080,725	Funding to projects that specified their activities to prevent and treat intentional self-injury.(9–11)
Interpersonal violence	6,769,823	Funding to projects that specified their activities targeting the prevention of dating violence, domestic violence, sexual assault, stalking, and also post-violence interventions among adolescents. (12–14)
Depressive disorders	6,708,125	Funding to projects that specified their activities to prevent, diagnose, or treat depressive disorder, including major depressive disorder and dysthymia. (1,15–17)
Low back and neck pain	6,016,741	Funding to projects that specified their activities to prevent, treat or relief low back and neck pain among adolescents, including the related structural, psychological, and genetic risk factors.(18,19)
Diarrheal diseases	5,924,662	Funding to projects that specified their activities to prevent, diagnose, or treat diarrhea diseases.(20,21)

© 2018 Li Z et al. *JAMA Network Open.*

**DALYs/Mortality causes/Risk factors**	**Total DALYs between 2003 and 2015 in the 132 studied countries**	**Definition**
Tuberculosis	5,037,705	Funding to projects that specified their activities to prevent, diagnose, or treat tuberculosis diseases, including drug-susceptible tuberculosis, multidrug0resistant tuberculosis without extensive drug resistance, extensively drug resistant tuberculosis, latent tuberculosis infection. (1,22–26)

© 2018 Li Z et al. *JAMA Network Open.*

eTable 6. Key Words Used to Search for Projects on Skin and Subcutaneous Diseases in the Creditor Reporting System

acantholytic disorder	acanthosis nigricans	acne	acne keloid	acquired epidermolysis bullosa	acquired ichthyosis
acquired keratoderma palmaris et plantaris	acquired keratosis palmaris et plantaris	acrodermatitis continua	actinic granuloma	actinic keratosis	actinic reticuloid
acute lymphadenitis	acute skin	ainhum	alopecia (capitis) totalis	alopecia areata	alopecia mucinosa
alopecia nos	alopecia totalis	anagen effluvium	androgenic alopecia	anetoderma	apocrine miliaria
atrophic disorder of skin	beau's lines	berloque dermatitis	besnier's prurigo	bromhidrosis	bullous disorder
café au lait spots	calcinosis cutis	candidid	capitis totalis	carbuncle	cellulitis
chloasma	chromhidrosis	chronic bullous disease of childhood	cicatricial alopecia	clubbed nail pachydermoperiostosis	connective tissue disorder
corns and callosities	cradle cap	cutaneous abscess	cutaneous autosensitization	cutis laxa senilis	cutis rhomboidalis nuchae
decubitus ulcer	dermatitis	dermatitis herpetiformis	dermatophytid	diaper rash	diminished melanin formation
drug photoallergic response	drug phototoxic response	duhring's disease	dyshidrosis	eccrine sweat disorder	eczema
eczematid	elastosis perforans serpiginosa	eosinophilic cellulitis	epidermal cyst	erythema	erythema elevatum diutinum
erythema intertrigo	erythematosus	erythematous	erythrasma	factitial dermatitis	febrile neutrophilic dermatosis
fogo selvagem	follicular cysts	follicular disorder	folliculitis decalvans	folliculitis ulerythematosa reticulata	fox-fordyce disease
freckles	furuncle	gianotti-crosti syndrome	gottron's papules	granuloma	granuloma faciale
granulomatous	hair color	hair colour	hair loss	hair shaft	hidradenitis suppurativa
hirsutism	hypertrichosis	impetigo	impetigo herpetiformis	infantile papular acrodermatitis	infections of skin and subcutaneous tissue
infective dermatitis	infiltrative disorder	ingrowing nail	juvenile dermatitis herpetiformis	keratoderma	keratosis follicularis et parafollicularis in cutem penetrans
keratosis punctata	lentigo	leukoderma	lichen nitidus	lichen planopilaris	lichen planus
lichen sclerosus et atrophicus	lichen simplex chronicus	lichen striatus	linear scleroderma	localized scleroderma	melanin hyperpigmentation
miliaria	morphea	mucha-habermann disease	mucinosis of skin	nail disorder	nail dystrophy

© 2018 Li Z et al. *JAMA Network Open.*

necrobiosis lipoidica	onychogryphosis	onycholysis	palmaris et plantaris	papulosis	papulosquamous disorder
papulosquamous disorder	parapsoriasis	paronychia	pemphigoid	pemphigus	perifolliculitis capitis abscedens
perioral dermatitis	photocontact dermatitis	pidermal thickening	pigmented purpuric dermatosis	pilar cyst	pilonidal cyst
pityriasis alba	pityriasis lichenoides chronica	pityriasis lichenoides et varioliformis acuta	pityriasis rosea	pityriasis rubra pilaris	poikiloderma of civatte
poikiloderma vasculare atrophicans	polymorphous light eruption	polytrichia	pompholyx	prurigo	pruritus
pseudofolliculitis barbae	pseudopelade	psoriasis	pustulosis palmaris et plantaris	pyoderma	pyoderma gangrenosum
pyogenic granuloma	radiodermatitis	reactive perforating collagenosis	rhinophyma	rosacea	scar
scar conditions	sclerodactyly	sebaceous cyst	seborrhoea capitis	seborrhoeic keratosis	senear-usher syndrome
skin appendage	skin changes	skin eruption	sneddon-wilkinson disease	solar urticaria	staphylococcal scalded skin syndrome
steatocystoma multiplex	stevens–johnson syndrome	striae atrophicae	subcorneal pustular dermatitis	sunburn	sweat disorder
telogen effluvium	transepidermal elimination	transepidermal elimination disorder	trichilemmal cyst	trichorrhexis nodosa	ulcer
urticaria	vasculitis	vitiligo	von zumbusch's disease	xerosis cutis	yellow nail syndrome

© 2018 Li Z et al. *JAMA Network Open.*

eTable 7. Key Words Used to Search for Road Injury Projects in the Creditor Reporting System

alcohol control study	alcohol testing	caring roadway	cyclist	head-on crash	helmet
helmet awareness raising	helmet wearing campaign	motorcyclist	pedestrian safety	railway safety	road accident
road across the street	road cooperative	road crash	road injuries	road management and safety project	road risk
road safety	safe system approach	safer road	school bus	school transport	seat belt use
speed compliance	traffic accident	traffic education	traffic injuries	transport among school children	transport improvement
transport services	transport services for boys and girls working in the streets	transportation safety	vehicle safety	young driver	

Note:

1. We have translated all the keywords listed above to Spanish, French, Portuguese, Italian, Dutch, German, Norwegian, and Swedish
2. We performed keywords searching using 1) all lower case; 2) all upper case; 3) capitalize the first letter of each word 4) capitalize the first letter of the term

© 2018 Li Z et al. *JAMA Network Open.*

eTable 8. Key Words Used to Search for HIV/AIDS Projects in the Creditor Reporting System
Projects funded with purpose name "STD control including hiv/aids".
Projects funded by Joint United Nations Programme on HIV/AIDS (UNAIDS).

Abstinence	acquired immune deficiency syndrome	acquired immunno deficiency syndrome	aids	aids education	antiretroviral therapy
ARV	ART	awareness	behavior change	behavior change communication	BCC
blood safety	care activities	care and support	cd4 count	chronically ill	clinical monitoring
condom	contraception	counselling and testing	diagnosis	drug regimens	essential service
Family planning	Global Fund to Fight AIDS	GFATM	haart	HAART	highly active antiretroviral therapy
hiv	hiv education	HIV	HIV/AIDS	human immunodeficiency virus	infected children
male circumcision	material support	microbicide	Millenuim Development Goal 6	MDG6	most at risk
mother to child aids transmission	mother to child hiv aids transmission	mother to child transmission	mother-to-child aids transmission	mother-to-child transmission	orphans
ovc	pain relief	parent to child transmission	parent-to-child transmission	people living with HIV/AIDS	PLHA
pmtct	PMTCT	prevent	prevention of mother-to-child transmission	psychological service	psychological support
psychosocial service	psychosocial support	reduce the transmission of hiv	reducing the transmission of hiv	retroviral	retrovirale
retroviralen	reverse transcriptase inhibitor	risky sexual behavior	safe blood supply	safe injection	social support
symptom relief	testing and counseling	testing and counselling	treat	tuberculosis	UNAIDS
vct	viral burden	viral load	viral titer	voluntary counselling and testing	VCT
vulnerable children	vulnerable group				

Note:

1. We have translated all the keywords listed above to Spanish, French, Portuguese, Italian, Dutch, German, Norwegian, and Swedish
2. We performed keywords searching using 1) all lower case; 2) all upper case; 3) capitalize the first letter of each word 4) capitalize the first letter of the term

© 2018 Li Z et al. *JAMA Network Open.*

eTable 9. Key Words Used to Search for Projects on Iron-Deficiency Anemia in the Creditor Reporting System

Anemia	beeturia	blood Transfusion	CBC	complete blood count	dietary iron
fatigue	ferrous sulfate	grains with iron	hee-MAT-oh-crit	hematocrit	heme iron
hemoatologic	hemoglobin	impaired absorption	intravenous iron	iron administration	iron deficiency
iron deficient	iron supplement	iron therary	iron-deficiency	iron-deficient	iron-rich protein
irritability	IV iron	non-heme iron	oral iron	pagophagia	pale
parenteral iron	pica	RBC	RBC transfusion	RBCs	red blood cell transfusion
red blood cells	restless	tired	transfusion	weakness	

Note:

1. We have translated all the keywords listed above to Spanish, French, Portuguese, Italian, Dutch, German, Norwegian, and Swedish
2. We performed keywords searching using 1) all lower case; 2) all upper case; 3) capitalize the first letter of each word 4) capitalize the first letter of the term

© 2018 Li Z et al. *JAMA Network Open.*

eTable 10. Key Words Used to Search for Self-Harm Projects in the Creditor Reporting System

CBT	cognitive behavioral therapy	cutting	DBT	dialectical behavioral therapy	eating disorder
hurt herself	hurt himself	hurt himself/herself	hurt themselves	hurt yourself	hurt yourselves
hurting herself	hurting himself	hurting himself/herself	hurting themselves	hurting yourself	hurting yourselves
intentional injury	intentional-injury	mental health professional	pharmacotherapy	psychatric	psychatrist
psychology	psychosis	psychotherapy	self burn	self harm	self hurt
self injuring	self injurious	self injury	self-burn	self-harm	self-hurt
self-injurers	self-injuring	self-injurious	self-injury	self-mutilation	SH

Note:

1. We have translated all the keywords listed above to Spanish, French, Portuguese, Italian, Dutch, German, Norwegian, and Swedish
2. We performed keywords searching using 1) all lower case; 2) all upper case; 3) capitalize the first letter of each word 4) capitalize the first letter of the term

© 2018 Li Z et al. *JAMA Network Open.*

eTable 11. Key Words Used to Search for Projects on Interpersonal Violence in the Creditor Reporting System

Assault	battering	beating	coercion	domestic violence	economic abuse
economic control	emotional abuse	frighten	gender based violence	gender empowerment	gender equality
gender project	girls empowerment	girls initiative programme	hitting	homicide	intimidating
IPV	isolation	kicking	male privilege	marginalized girls	physical abuse
self-esteem	sexual abuse	sexual violence	slapping	stalking	threat
UN programme on adolescent girls	violence	violence and injuries	violence sexuelle	violent	youth gender and equality network

Note:

1. We have translated all the key words listed above to Spanish, French, Portuguese, Italian, Dutch, German, Norwegian, and Swedish
2. We performed key words searching using 1) all lower case; 2) all upper case; 3) capitalize the first letter of each word 4) capitalize the first letter of the term

© 2018 Li Z et al. *JAMA Network Open.*

eTable 12. Key Words Used to Search for Projects on Depressive Disorders in the Creditor Reporting System

amitriptyline	antidepressant	antipsychotic	anxious distress	aplenzin	atypical depression
bipolar	bupropion	catatonia	celexa	citalopram	cyclothymic disorder
decreased interest	depressive disorder	desipramine	doxepin	DSM-5	dysphoric disorder
dysthymia	ECT	electroconvulsive therapy	emotion	emsam	escitalopram
fluoxetine	forfivo XL	imipramine	isocarboxazid	lexapro	MAOIs
marplan	MDD	mental health professional	mirtazapine	mood	nardil
nefazodone	norpramin	nortriptyline	pamelor	parnate	paroxetine
paxil	peripartum depression	pexeva	phenelzine	PMDD	postpartum depression
premenstrual dysphoric disorder	protriptyline	prozac	psychiatric evaluation	psychiatrist	psychologist
psychotherapy	psychotic depression	remeron	SAD	sadness	seasonal affective disorder
selective serotonin reuptake inhibitor	selegiline	serotonin-norepinephrine reuptake inhibitor	sertraline	situational depression	situational' depression
SNRI	SSRI	surmontil	TMS	tofranil	transcranial magnetic stimulation
tranylcypromine	trazodone	trimipramine	trintellix	viibryd	vilazodone
vivactil	vortioxetine	wellbutrin SR	wellbutrin XL	zoloft	

Note:

1. We have translated all the key words listed above to Spanish, French, Portuguese, Italian, Dutch, German, Norwegian, and Swedish
2. We performed key words searching using 1) all lower case; 2) all upper case; 3) capitalize the first letter of each word 4) capitalize the first letter of the term

eTable 13. Key Words Used to Search for Projects on Lower Back and Neck Pain in the Creditor Reporting System

autoimmun	back muscle	cervical degenerative disc disease	cervical osteoarthritis	cervical spine	cold therapy
compression fracture	deformity	degenerative disc disease	endorphin	exercise core	facet joint osteoarthritis
foraminal stenosis	gland swelling	hamstring	heat therapy	herniated disc	inner endorphin
isthmic spondylolisthesis	ligament sprain	low back	lower back	lower back and neck pain	lower back muscle
lumbar disc herniation	lumbar herniated disc	lumbar Spine	lymph node (gland) swelling	lymph node swelling	musclestrain
neck injury	neck pain	neck strain	osteoarthritis	osteomyelitis	pinched nerve
pulled back muscle	restorative sleep	sacroiliac joint dysfunction	spinal stenosis	spondylolisthesis	stenosis with myelopathy
tumor	virus infection of the throat	whiplash			

Note:

1. We have translated all the key words listed above to Spanish, French, Portuguese, Italian, Dutch, German, Norwegian, and Swedish
2. We performed key words searching using 1) all lower case; 2) all upper case; 3) capitalize the first letter of each word 4) capitalize the first letter of the term

© 2018 Li Z et al. *JAMA Network Open.*

eTable 14. Key Words Used to Search for Diarrheal Projects in the Creditor Reporting System

access to water	anti-diarrheal	antidiarrheal	bacteriological	boil	borehole
bottled water	cart with small drum	cart with small tank	chemical Purification	chlorinated water	chlorine drop
chlorine treatment	clean water	composting toilet	contaminated water	crystal	dehydration
depth Filter	diarrhea	diarrhoea	drink liquid	drinking liquid	drinking water
hand washing	handwashing	hygiene	hypochlorite	hypochlorite solution	improved drinking water
improved water	iodine crystal	iodine solution	iodine treatment	Katadyn	lack of water
latrine	membrane filter	MSR WaterWorks	norovirus	oral rehydration solution	ORS
OTC	Over-the-counter	piped sewer system	piped water	pit latrine	poor hygiene
potable aqua	protected dug wells	protected spring	public tap	PUR Scout	PUR-Hiker
rainwater collection	rehydration drink	rotavirus	safe container	safe water	safe water
septic system	soap	sodium hypochlorite	standpipe	surface water	tablet
tanker-truck	toxic Water	tubewell	ultraviolet	unclean water	unimproved sanitation
unsae drinking water	unsafe water	vendor provided water	washhand	waste water	wastewater
water disinfection	water epidemic	water filter	water purification	water purifier	water quality
water storage	WASH				

Note:

1. We have translated all the key words listed above to Spanish, French, Portuguese, Italian, Dutch, German, Norwegian, and Swedish
2. We performed key words searching using 1) all lower case; 2) all upper case; 3) capitalize the first letter of each word 4) capitalize the first letter of the term

© 2018 Li Z et al. *JAMA Network Open.*

eTable 15. Key Words Used to Search for Tuberculosis Projects in the Creditor Reporting System

amikacin	anti lepre-tbc	anti-lepre et tbc	anti-TB	ARV treatment through DOTS and MTCT prevention	bacillus calmette-guerin
BCG	bedaquiline	capreomycin	directly observed treatment	DOTS	DR-TB
drug resistant TB	drug resistant tuberculosis	drug-resistant tuberculosis	drug-resistent TB	drug-susceptible TB	drug-susceptible tuberculosis
EPTB	ethambutol	fluoroquinolones	INH	IPT	isoniazid
isoniazid prevention therapy	kanamycin	linezolid	LTBI	myambutol	pyrazinamide
rifadin	rifampin	rimactane	TB	TB-DOTS	TBC
tubercullosis	tuberculose	tuberculosis			

Note:

1. We have translated all the key words listed above to Spanish, French, Portuguese, Italian, Dutch, German, Norwegian, and Swedish
2. We performed key words searching using 1) all lower case; 2) all upper case; 3) capitalize the first letter of each word 4) capitalize the first letter of the term

© 2018 Li Z et al. *JAMA Network Open.*

eFigure 1. The Proportion of DAAH (Adolescent Targeted) in DAH (%), 2003-2015[1, 2]

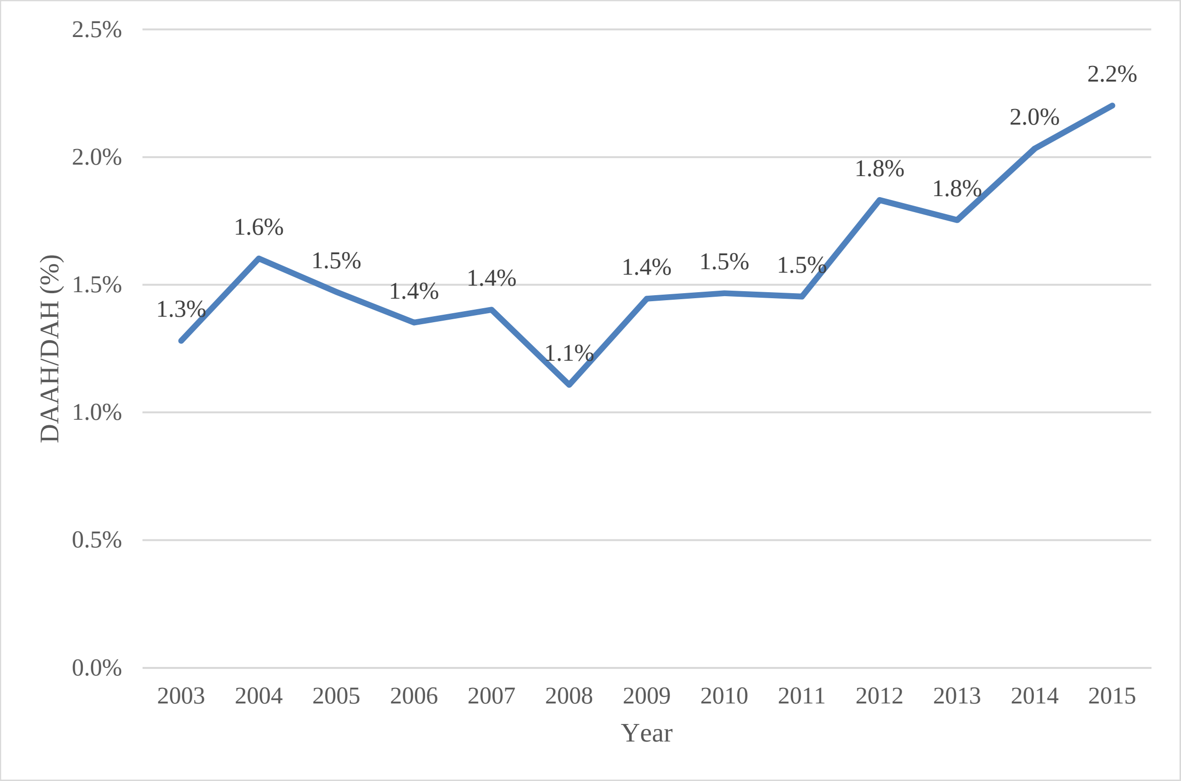

Note:

1. "DAAH" represents "development assistance for adolescent health"; "DAH" represents "development assistance for health".
2. The DAH disbursement values based on the authors' estimation including all projects with the sector code 120 (Health, Total), 121 (Health, General), 122 (Basic Health), and 130 (Population policies/programmes & Reproductive Health).

© 2018 Li Z et al. *JAMA Network Open.*

eTable 16. Annual DAAH (Adolescent Targeted) and DAH Disbursement ($ million), 2003-2015[1, 2]

**year**	**DAAH disbursement ($ million)**	**DAH ($ million)**
2003	109.7	8,566.5
2004	136.6	8,520.9
2005	159.6	10,845.1
2006	162.8	12,040.6
2007	191.9	13,690.7
2008	169.9	15,333.3
2009	276.0	19,097.6
2010	312.3	21,291.4
2011	309.1	21,270.8
2012	394.6	21,541.3
2013	422.4	24,090.5
2014	461.2	22,676.9
2015	528.5	24,008.5

Note:

1. "DAAH" represents "development assistance for adolescent health"; "DAH" represents "development assistance for health".
2. The DAH disbursement values based on the authors' estimation including all projects with the sector code 120 (Health, Total), 121 (Health, General), 122 (Basic Health), and 130 (Population policies/programmes & Reproductive Health).

© 2018 Li Z et al. *JAMA Network Open.*

eFigure 2. Trends in Annual DAAH (Adolescent Inclusive) and Annual SRH or HIV/AIDS Disbursement, 2003-2015 (million US$)

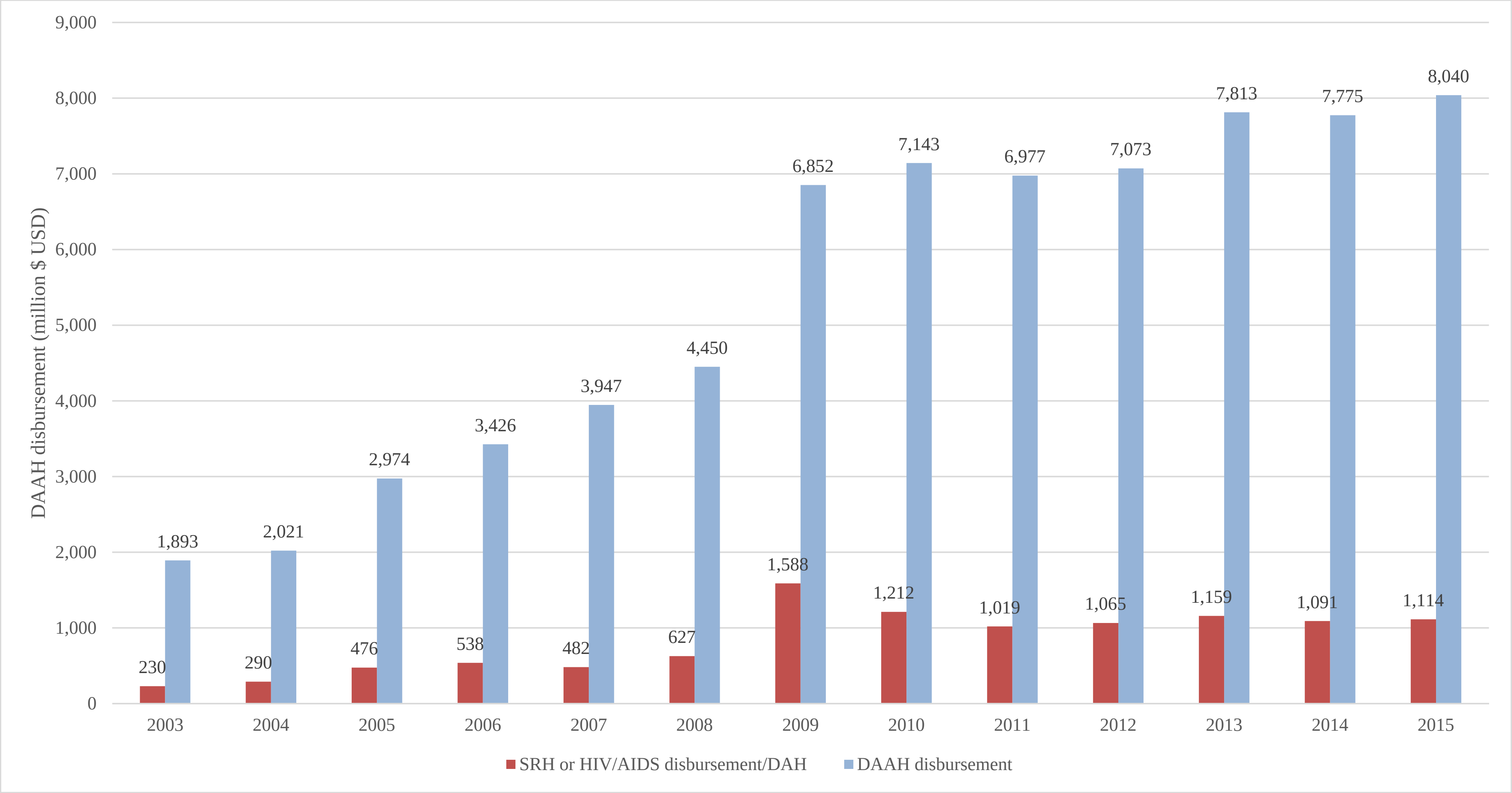

Note:

1. All disbursement data were converted to constant 2015 US$

© 2018 Li Z et al. *JAMA Network Open.*

eFigure 3. Annual DAAH (Adolescent Targeted) by Donors, 2003-2015 (million US$)

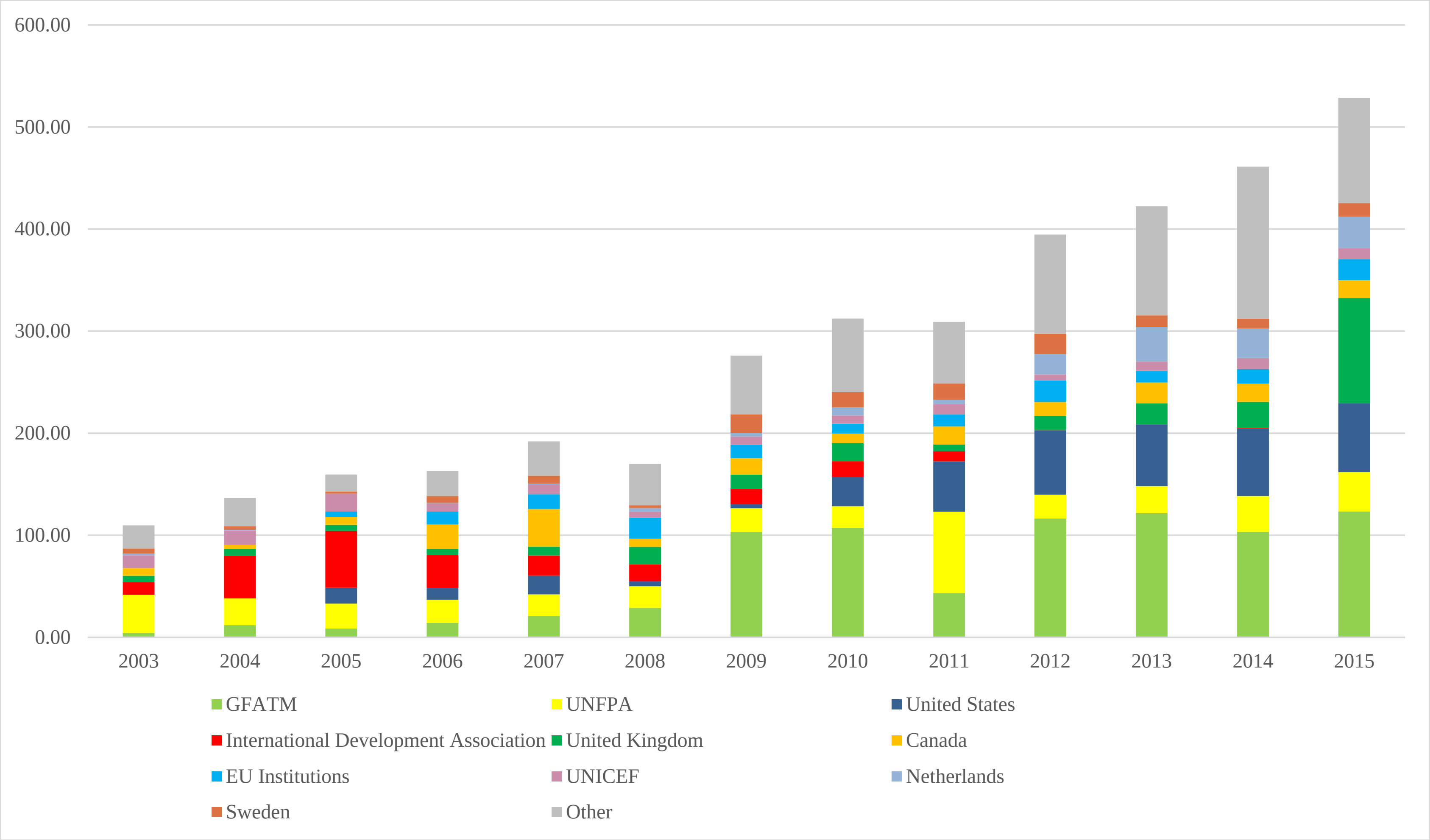

Note:

1. "GFATM" represents "Global Fund to Fight AIDS, Tuberculosis and Malaria"
2. "UNFPA" represents United Nations Population Fund
3. All disbursement data were converted to constant 2015 US$

© 2018 Li Z et al. *JAMA Network Open.*

eFigure 4. Annual DAAH (Adolescent Inclusive) by Donors, 2003-2015 (million US$)

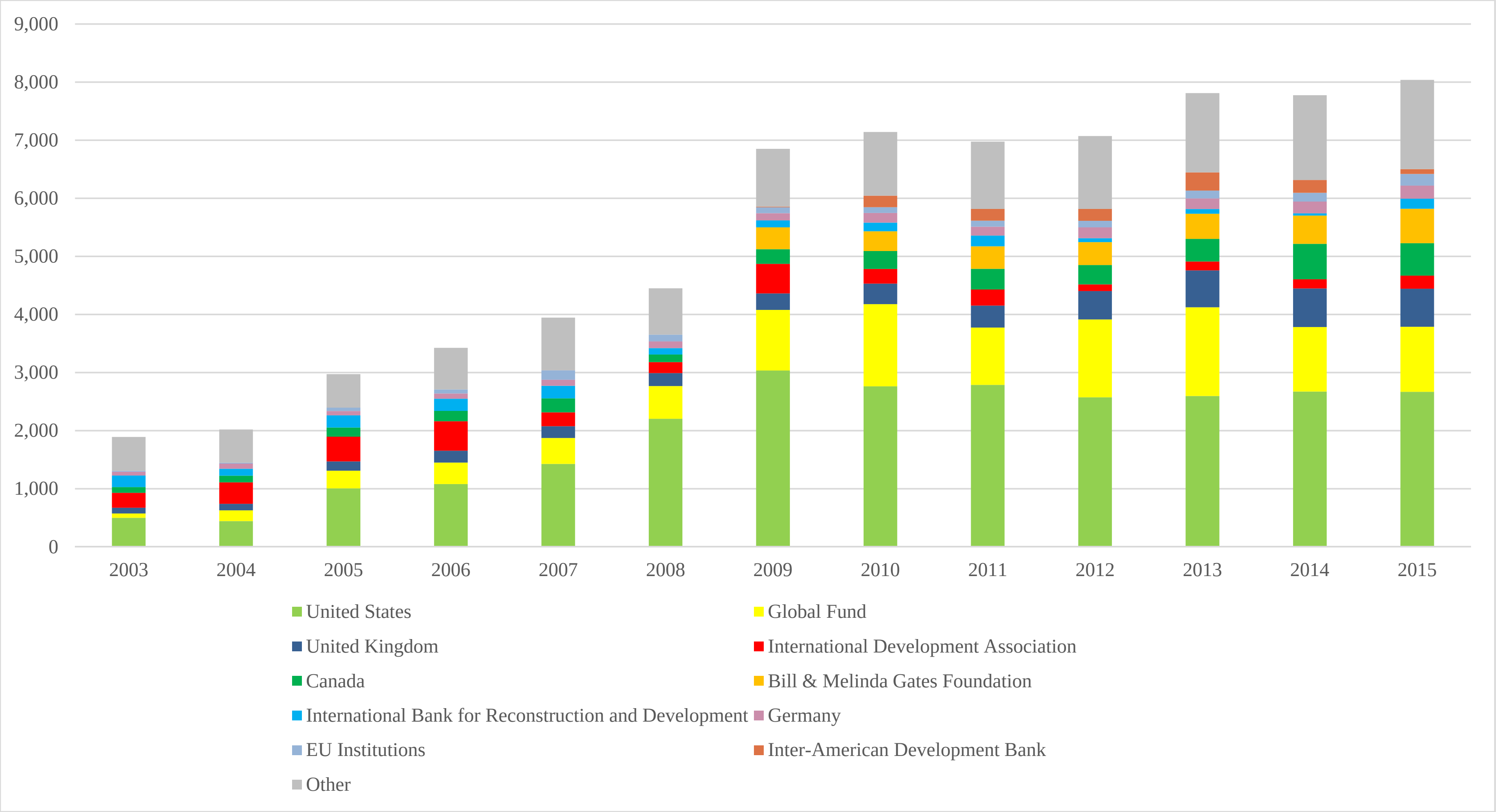

Note:

4. "GFATM" represents "Global Fund to Fight AIDS, Tuberculosis and Malaria"
5. "UNFPA" represents United Nations Population Fund
6. All disbursement data were converted to constant 2015 US$

© 2018 Li Z et al. *JAMA Network Open.*

eTable 17. DAAH (Adolescent Inclusive) per Adolescent (US$) by Recipient Region and Income Classification, 2003-2015

	Adolescent-inclusive DAAH per adolescent (US$)													Annual growth rate (%)
	**2003**	**2004**	**2005**	**2006**	**2007**	**2008**	**2009**	**2010**	**2011**	**2012**	**2013**	**2014**	**2015**	
**Overall**														
Overall	0.95	1.07	1.48	1.66	1.81	2.20	3.31	3.44	3.26	3.31	3.70	3.68	3.75	12.09%
LICs	2.51	2.86	3.52	4.25	4.28	5.63	8.77	9.59	8.35	9.07	9.73	10.02	9.97	12.17%
LMCs	0.76	0.94	1.43	1.49	1.67	1.88	2.85	2.55	2.46	2.38	2.75	2.68	2.93	11.85%
UMCs	0.60	0.55	0.77	0.90	1.03	1.21	1.62	1.94	2.02	1.83	2.01	1.79	1.56	8.26%
**Recipient region**														
Africa	2.53	3.30	4.04	5.10	5.02	6.74	9.78	10.01	8.88	10.11	10.99	10.61	10.57	12.65%
SSA	2.65	3.45	4.22	5.32	5.23	7.01	10.16	10.38	9.19	10.45	11.34	10.92	10.87	12.48%
LICs	3.27	3.98	4.72	5.90	5.75	7.12	10.79	11.87	10.13	11.85	12.34	12.25	12.40	11.76%
LMCs	1.78	2.63	3.43	4.35	3.86	5.57	7.39	6.88	6.66	7.06	9.07	8.01	8.18	13.56%
UMCs	1.47	2.19	2.72	3.58	5.15	8.73	12.63	10.79	9.69	11.07	9.97	10.38	8.20	15.43%
Americas	1.85	1.34	2.04	2.43	2.77	2.84	3.69	4.83	4.77	3.89	4.78	3.40	3.81	6.22%
LICs	3.53	4.46	7.38	12.25	17.66	21.02	31.62	25.05	28.62	25.03	26.45	24.62	27.35	18.61%
LMCs	3.91	3.53	5.37	6.04	7.08	9.46	12.79	13.04	11.64	9.23	10.54	7.03	9.80	7.95%
UMCs	1.59	1.04	1.55	1.81	1.95	1.67	2.01	3.42	3.38	2.75	3.58	2.46	2.52	3.93%
Eastern Mediterranean	0.78	0.68	2.05	1.52	2.40	2.38	5.00	4.32	3.71	3.32	3.50	4.24	3.95	14.47%
LICs	0.74	0.45	3.97	0.85	2.02	9.64	23.48	24.86	19.32	11.41	10.11	16.23	12.81	35.56%
LMCs	0.78	0.65	1.79	1.70	2.74	1.90	4.12	2.81	2.30	2.32	2.59	2.73	3.03	12.01%
UMCs	0.80	0.82	2.41	1.11	1.32	2.05	2.78	3.69	4.07	4.44	4.76	5.84	4.13	14.68%
Europe	0.89	0.91	1.33	1.99	2.35	2.85	4.07	3.91	5.07	3.91	3.65	6.62	6.30	17.76%
LICs	1.99	1.74	2.46	1.89	3.05	4.38	4.93	6.60	7.55	8.84	8.82	7.86	6.84	10.86%
LMCs	0.66	0.78	1.77	2.26	3.42	3.27	5.51	4.46	5.18	5.30	4.24	12.72	13.34	28.43%
UMCs	0.84	0.86	0.87	1.81	1.51	2.36	2.95	3.18	4.66	2.33	2.58	2.61	1.89	7.00%

© 2018 Li Z et al. *JAMA Network Open.*

	Adolescent-inclusive DAAH per adolescent (US$)													Annual growth rate (%)
	**2003**	**2004**	**2005**	**2006**	**2007**	**2008**	**2009**	**2010**	**2011**	**2012**	**2013**	**2014**	**2015**	
South-East Asia	0.50	0.62	0.75	0.69	0.67	0.79	1.09	1.15	1.16	0.90	1.08	1.04	1.05	6.26%
LICs	1.25	1.04	1.01	1.22	1.05	1.49	1.91	2.34	2.12	1.91	2.99	3.29	2.95	7.40%
LMCs	0.37	0.54	0.70	0.57	0.58	0.66	0.93	0.91	0.93	0.70	0.69	0.61	0.72	5.64%
UMCs	0.44	0.77	0.77	1.61	1.28	1.22	1.56	2.25	3.08	1.60	2.49	2.00	0.78	4.88%
Western Pacific	0.29	0.33	0.42	0.43	0.51	0.52	0.79	0.72	0.80	0.92	0.85	0.77	0.88	9.64%
LICs	3.71	3.29	5.31	5.12	6.16	7.14	11.18	10.22	9.44	7.32	8.77	10.26	11.17	9.63%
LMCs	1.18	1.07	1.67	1.65	1.77	1.97	2.96	2.84	2.94	3.40	3.69	3.15	3.51	9.53%
UMCs	0.09	0.16	0.12	0.15	0.20	0.17	0.25	0.20	0.18	0.23	0.12	0.10	0.11	1.35%

Note:

1. We didn’t include unallocable or regional DAAH projects
2. All disbursement data were converted to constant 2015 US$
3. We calculated DAAH per adolescent for each category with the formula: Total DAAH disbursements to all countries in that category/ Total population of all countries in that category. For example, DAAH per adolescent in Africa=The sum of DAAH to each African country/ The sum of adolescent population in each African country. If a country is with missing data on adolescent population or DAAH disbursement, we excluded the country from our analysis.
4. “LICs” represents “Low-income countries”; “LMCs” represents “Lower-middle income countries”; “UMCs” represents “Upper-middle income countries”. We base on 2010 World Bank income classification.
5. Annual growth rate=((end value/start value)^(1/periods) -1.

© 2018 Li Z et al. *JAMA Network Open.*

eTable 18. DAAH (Adolescent Targeted) per Adolescent (US$) by Recipient Country, 2003-2015

WHO region	Recipient country	Adolescent-targeted DAAH per adolescent (US$)													Annual growth rate (%)
		2003	2004	2005	2006	2007	2008	2009	2010	2011	2012	2013	2014	2015	
Africa	Algeria	0.00	0.01	0.01	0.02	0.00	0.04	0.01	0.02	0.01	0.04	0.02	0.02	0.00	-0.97%
	Angola	0.10	0.12	0.09	0.03	0.11	0.05	0.02	1.62	0.48	0.41	1.29	0.02	0.03	-9.27%
	Benin	0.19	0.13	0.19	0.14	0.18	0.16	0.29	0.10	0.48	0.21	0.22	0.30	0.19	0.32%
	Botswana	2.79	0.58	0.38	0.36	0.29	0.45	0.80	0.69	1.80	0.45	0.34	0.11	0.20	-19.78%
	Burkina Faso	0.11	0.09	0.09	0.20	0.13	0.10	0.17	0.49	0.25	0.11	0.14	0.13	0.12	1.15%
	Burundi	0.13	0.17	0.22	0.07	0.21	0.16	0.13	0.83	1.32	0.40	0.45	1.27	1.47	22.26%
	Cabo Verde	0.97	0.64	0.94			15.02	0.24	0.25	0.00	0.01	0.04	58.59	6.00	16.36%
	Cameroon	0.10	0.20	0.17	0.07	0.21	0.05	0.22	0.17	0.14	0.08	0.05	0.08	0.33	10.01%
	Central African Republic		0.10	0.27	0.14	0.07	0.01	0.72		0.02	0.17	0.12	0.03	0.20	6.77%
	Chad	0.04	0.18	0.08	0.01	0.01	0.10	0.12	0.47	0.09	0.02	0.02	0.05	0.11	9.78%
	Comoros	0.42	2.56	0.20	1.09	0.83	1.10	0.26	2.90	3.54	3.60	1.41	2.59	2.32	15.19%
	Congo	0.04	0.12	0.03	0.28	0.13	0.36	0.09	0.03	0.80	0.31	0.41	0.70	0.50	23.75%
	Côte d'Ivoire	0.06	0.09	0.03	0.04	0.16	0.11	0.08	0.47	0.39	0.05	0.11	0.10	0.11	5.58%
	Democratic Republic of the Congo	0.10	0.32	0.30	0.28	0.27	0.07	0.04	0.06	0.39	0.11	0.01	0.27	0.35	11.08%
	Djibouti		0.00	2.89	0.10	0.34	0.21	0.17	0.38	0.06	0.01	0.28	0.30	0.17	44.85%
	Equatorial Guinea		0.04		1.45					0.02	0.35		0.64	2.65	47.90%

© 2018 Li Z et al. *JAMA Network Open.*

WHO region	Recipient country	Adolescent-targeted DAAH per adolescent (US$)													Annual growth rate (%)
		2003	2004	2005	2006	2007	2008	2009	2010	2011	2012	2013	2014	2015	
Africa	Eritrea				0.00	0.02	0.00	3.95	4.82		4.79	3.13	0.60	0.13	59.88%
	Ethiopia	0.06	0.10	0.09	0.11	0.24	0.26	0.35	0.39	0.43	0.40	0.37	0.19	0.39	17.36%
	Gabon	0.78	0.80	0.48	0.12	0.05	0.10	0.12	0.14	0.07	0.02	0.07	0.07	0.59	-2.26%
	Gambia	0.44	0.51	0.29	0.49	0.33	0.26	0.13	0.13	0.12	0.36	0.00	0.04	0.08	-12.92%
	Ghana	0.16			0.03	0.04	0.03	0.60	0.12	0.28	0.55	1.34	1.35	1.49	20.70%
	Guinea	0.07	0.14	0.08	0.13	0.01	0.02	0.08	0.08	0.52	0.45	0.36	0.01	0.20	8.65%
	Guinea-Bissau	0.29	0.55	0.72	0.57	0.45	0.98	0.61	0.62	0.54	0.92	0.96	0.36	0.69	7.52%
	Kenya	0.30	0.45	0.37	0.34	0.32	0.37	0.51	0.51	0.38	0.52	0.31	0.46	0.46	3.57%
	Lesotho	0.17	0.14	0.08	0.01	0.55	0.2	5.97	8.00	2.19	3.66	4.02	4.89	1.08	16.80%
	Liberia	0.07	0.00	0.01	0.06	0.03	0.09	1.82	0.81	2.62	0.51	1.73	1.01	1.89	31.58%
	Madagascar	0.05	0.04	0.04	0.13	0.06	0.22	0.06	4.11	0.83	2.38	0.66	0.14	0.26	15.33%
	Malawi	0.63	0.57	0.42	0.58	0.51	1.19	0.52	0.24	0.27	11.48	9.80	11.76	17.46	31.91%
	Mali	0.08	0.19	0.13	0.09	0.39	0.14	0.35	0.45	0.82	0.19	0.16	0.31	0.64	18.94%
	Mauritania	0.48	0.49	0.48	0.50	0.51	1.02	0.02	0.01	0.10	0.14	0.06	0.19	0.40	-1.41%
	Mauritius	0.29	0.08	0.04	0.02	0.02	0.13	0.13	0.11	0.04	0.12	0.03	0.15	0.16	-4.95%
	Mozambique	1.34	0.64	0.32	0.33	1.39	0.81	1.23	0.61	1.20	1.27	0.90	0.33	0.36	-10.28%
	Namibia	0.92	0.45	0.43	0.75	0.61	0.23	0.76	0.30	1.04	0.69	0.33	0.35	0.32	-8.47%
	Niger	0.15	0.68	0.30	0.40	0.65	0.20	0.18	0.27	0.49	0.05	0.08	1.09	0.81	15.41%

© 2018 Li Z et al. *JAMA Network Open.*

WHO region	Recipient country	Adolescent-targeted DAAH per adolescent (US$)													Annual growth rate (%)
		**2003**	**2004**	**2005**	**2006**	**2007**	**2008**	**2009**	**2010**	**2011**	**2012**	**2013**	**2014**	**2015**	
Africa	Nigeria	0.03	0.03	0.05	0.02	0.03	0.03	0.04	0.06	0.07	0.04	0.01	0.01	0.09	8.77%
	Rwanda	0.17	0.23	0.07	0.14	0.46	0.31	6.71	0.74	0.72	0.25	0.32	0.16	2.77	26.43%
	Sao Tome and Principe	0.00				0.09	7.51		0.01	2.76	6.93	4.99	34.5	1.36	149.40%
	Senegal	1.30	2.69	3.16	1.47	0.40	0.22	0.32	0.22	0.17	0.23	0.15	0.40	0.76	-4.34%
	Seychelles	2.06	0.84	0.67					0.12	3.26	1.44	0.02			-35.94%
	Sierra Leone	0.73	3.86	4.49	4.90	4.76	2.26	0.93	2.04	2.23	4.99	4.15	3.15	3.71	14.52%
	Somalia		0.07	0.09	0.06	0.06	0.02	0.08	0.15	0.33	0.4	0.48	1.01	0.47	19.28%
	South Africa	0.04	0.02	0.00	0.01	0.02	0.13	0.53	0.07	0.08	0.15	0.17	0.46	0.36	20.88%
	South Sudan									2.04	2.08	2.10	5.67	5.60	28.69%
	Sudan			0.00	0.01	0.01	0.03	0.04	0.33	0.54	0.35	0.20	0.14	0.04	27.62%
	Swaziland	0.01	0.08		0.38	0.57	0.54	0.64	0.76	2.82	0.19	0.02	0.15	0.48	37.68%
	Togo	0.09	0.18	0.09	0.13	0.12	0.17	2.54	1.29	0.14	0.16	0.11	0.63	0.23	8.08%
	Uganda	0.50	0.10	0.13	0.12	0.10	0.11	0.08	0.07	0.07	0.13	0.41	0.55	0.82	4.12%
	United Republic of Tanzania	0.35	0.28	0.32	0.28	0.35	0.23	0.28	0.48	0.67	0.66	0.94	0.85	0.36	0.17%
	Zambia	0.10	0.07	0.06	0.13	0.08	0.30	0.13	0.08	0.38	0.44	1.41	2.25	0.94	20.30%
	Zimbabwe	0.47	0.22	0.11	0.45	0.13	0.26	0.31	0.29	0.54	0.47	0.51	0.66	0.72	3.65%

© 2018 Li Z et al. *JAMA Network Open.*

WHO region	Recipient country	Adolescent-targeted DAAH per adolescent (US$)													Annual growth rate (%)
		**2003**	**2004**	**2005**	**2006**	**2007**	**2008**	**2009**	**2010**	**2011**	**2012**	**2013**	**2014**	**2015**	
Americas	Argentina	0.23		0.01	0.01	0.01	0.02	0.04	0.04	0.02	0.02	0.01	0.02	0.08	-8.05%
	Belize	2.48	2.93	4.80	1.16	0.57	0.08	4.14	9.60	3.46	13.14	13.38	1.95	8.43	10.74%
	Bolivia	0.23	0.42	0.1	1.03	0.57	0.72	0.82	0.21	1.02	0.67	0.99	0.75	0.96	12.60%
	Brazil	0.00	0.00	0.01	0.01	0.02	0.02	0.02	0.02	0.01	0.01	0.01	0.03	0.02	13.14%
	Chile	0.03	0.03	0.07	0.02	0.10	0.03	0.01	0.03	0.07	0.04	0.18	0.06	0.01	-10.18%
	Colombia	0.05	0.05	0.02	0.04	0.07	0.17	0.20	0.05	0.05	0.07	0.12	0.08	0.12	8.38%
	Costa Rica	0.17	0.20	0.18	0.11	0.01	0.00	0.15	0.27	0.62	0.21	0.27	0.01	0.19	0.84%
	Cuba	0.17	0.20	0.20	0.11	0.1	0.41	0.29	1.70	0.86	1.30	3.79	1.01	3.53	28.88%
	Dominican Republic	0.04	0.08	0.11	0.27	0.19	0.34	0.25	0.14	0.26	0.03	0.05	0.08	0.13	9.91%
	Ecuador	0.30	0.17	0.37	0.29	0.36	0.06	0.23	0.85	0.83	0.50	1.16	1.13	1.14	11.67%
	El Salvador	0.21	0.28	0.69	0.44	1.00	1.61	1.53	2.14	2.37	1.72	2.66	3.15	3.10	25.10%
	Grenada							11.35	3.51	13.47	1.72	3.56	2.26	3.63	-17.31%
	Guatemala	0.16	0.03	0.15	0.15	0.20	0.18	0.69	1.68	0.98	1.47	1.52	1.92	0.32	5.70%
	Guyana	13.07	6.18	10.74	30.76	24.32	0.24	0.03		0.16	0.02	0.05	0.05	0.28	-27.30%
	Haiti	0.09	0.04	0.37	1.52	3.44	0.13	8.93	0.57	0.68	0.25	0.38	0.54	0.29	9.94%
	Honduras	0.24	0.23	0.16	0.15	0.10	1.13	0.57	0.93	2.05	1.46	2.42	1.76	1.37	15.66%
	Jamaica	0.65	0.44	4.22	4.66	5.85	2.31	0.60	0.16	0.16	0.04	0.00	1.54	2.12	10.36%
	Mexico	0.01	0.00	0.00	0.00	0.01	0.01	0.02	0.01	0.01	0.02	0.01	0.03	0.10	20.47%

© 2018 Li Z et al. *JAMA Network Open.*

WHO region	Recipient country	Adolescent-targeted DAAH per adolescent (US$)													Annual growth rate (%)
		**2003**	**2004**	**2005**	**2006**	**2007**	**2008**	**2009**	**2010**	**2011**	**2012**	**2013**	**2014**	**2015**	
America	Nicaragua	0.79	0.61	0.97	0.57	0.82	1.45	1.53	2.48	3.50	2.37	1.44	1.15	0.79	0.02%
	Panama	0.12	0.28	0.50	0.32	0.05	0.00	0.20	0.11		0.13	0.13	0.15	0.32	8.57%
	Paraguay	0.02	0.03	1.14	0.11	0.09	0.46	1.38	1.16	0.15	0.32	0.22	0.24	0.10	14.59%
	Peru	0.15	0.08	0.04	0.11	0.18	0.30	0.19	0.24	0.10	0.18	0.10	0.10	0.22	3.12%
	Saint Vincent and the Grenadines							0.13	0.91	0.46	0.13	0.02			-37.81%
	Suriname				0.08				0.17			0.07	0.02		-17.35%
	Uruguay	0.19	0.10	0.15	0.06	0.12	0.39	0.12	0.34	0.17	0.08	0.16	0.08	0.21	0.61%
	Venezuela (Bolivarian Republic of)	0.03	0.05	0.05	0.06	0.02	0.02	0.05	0.03	0.03	0.06	0.11	0.11	0.04	2.89%
Eastern Mediterranean	Afghanistan		0.02	0.17	0.01	0.03	0.01	0.16	0.36	0.96	0.23	0.21	0.58	0.86	39.52%
	Egypt	0.02	0.02	0.02	0.01	0.02	0.03	0.02	0.07	0.09	0.19	0.11	0.04	0.12	19.17%
	Iran (Islamic Republic of)	0.02		0.01	0.00	0.00	0.00	0.01	0.01	0.01	0.01	0.01	0.01	0.01	-9.86%
	Iraq	0.00	0.01		0.01	0.00	0.20	0.31	0.13	0.07	0.07	0.08	0.07	0.17	38.17%
	Jordan	0.18	0.13	0.16	0.14	0.06	0.04	0.29	0.88	0.19	0.15	0.28	0.20	0.37	6.12%
	Lebanon	0.97	0.78	0.09	1.17	1.50	1.22	1.52	0.54	1.41	1.13	0.58	0.51	0.70	-2.65%
	Libya												0.26	0.02	-91.76%
	Morocco	0.05	0.07	0.10	0.27	0.15	0.04	0.64	0.68	0.33	0.41	0.14	0.12	0.07	2.64%
	Pakistan	0.00	0.00	0.00	0.00	0.00	0.01	0.00	0.00	0.01	0.02	0.02	0.01	0.00	1.69%
	Syrian Arab Republic		0.01	0.00	0.01	0.01	0.01	0.01	0.02	0.02	0.01	0.01	0.02	0.05	20.44%
	Tunisia	0.09	0.08	0.06	0.06	0.04	0.05	0.09	0.04	0.01	0.06	0.04	0.65	0.52	15.49%
	Yemen	0.67	1.20	0.10	0.04	0.02	0.08	0.26	0.68	0.17	0.94	0.16	0.05	0.04	-20.40%

© 2018 Li Z et al. *JAMA Network Open.*

**WHO region**	**Recipient country**	**Adolescent-targeted DAAH per adolescent (US$)**													**Annual growth rate (%)**
		**2003**	**2004**	**2005**	**2006**	**2007**	**2008**	**2009**	**2010**	**2011**	**2012**	**2013**	**2014**	**2015**	
Europe	Albania	0.33	0.28		0.63	0.62	0.23	0.71	0.13	0.42	0.34	0.27	0.62	0.90	8.82%
	Armenia		0.11	0.30	0.24	0.04	0.09	0.03	0.00	0.73	0.16	0.26	0.08	0.16	3.46%
	Azerbaijan		0.02		0.04	0.06	0.08	0.24	0.03	0.21	0.09	0.23	0.08	0.15	20.82%
	Belarus			0.27	0.18	0.17	0.60	0.95	0.30	0.55	0.25	0.05	0.06	0.12	-8.06%
	Bosnia and Herzegovina	0.48	0.31	0.72	2.22	4.93	3.03	2.51	6.93	8.54	4.05	10.57	6.52	5.39	22.40%
	Georgia	0.22	0.21	0.07	0.98	1.03	1.04	0.50	0.12	1.29	2.00	1.65	1.52	1.08	14.03%
	Kazakhstan	0.17	0.69	0.70	1.35	0.68	1.26	0.06	0.03	0.13	0.18	0.04	0.06	0.08	-6.47%
	Kyrgyzstan	0.03	0.08	0.12	0.07	0.07	0.07	0.05	0.29	0.30	0.68	1.20	1.47	0.90	31.96%
	Mayotte								2.89						
	Moldova	1.23	1.88	0.11	2.51	9.21	4.81	1.34	4.97	4.25	1.67	0.76	1.43	1.74	2.95%
	Montenegro				1.27	1.03	3.55					0.01	0.02	0.50	-9.87%
	Serbia	0.27	0.13	0.29	0.33	0.46	0.17	0.85	0.12	0.70	0.22	0.14	0.12	0.04	-14.10%
	Tajikistan	0.39	0.49	0.52	0.08	0.10	0.25	0.02	0.19	0.16	0.06	0.04	0.03	0.04	-16.90%
	The former Yugoslav Republic of Macedpnia	0.18	0.04	0.53	0.72	0.80	0.42		0.07	0.23	0.20	0.51	0.43	0.20	1.11%
	Turkey	0.02	0.01	0.02	0.01	0.02	0.01	0.01	0.01	0.04	0.01	0.02	0.01	0.01	-10.52%
	Turkmenistan		0.06	0.15	0.13	0.10	0.05	0.06	0.04	0.05	0.08	0.07	0.08	0.08	2.32%
	Ukraine			0.04	0.05	0.08	0.17	0.13	0.23	0.15	0.34	0.14	0.20	0.45	25.94%
	Uzbekistan	0.01	0.02	0.12	0.03	0.02	0.04	0.67	0.23	0.07	0.02	0.03	0.02	0.01	1.25%
South-East Asia	Bangladesh	0.04	0.08	0.09	0.08	0.16	0.33	0.23	0.05	0.05	0.19	0.60	0.99	0.31	19.94%
	Bhutan	0.14			0.01	0.01	2.91	2.14	3.77	2.79	2.54	2.39	1.56	0.53	11.67%
	Democratic People's Republic of Korea												0.00	0.00	-9.56%
	India	0.01	0.04	0.07	0.04	0.02	0.01	0.01	0.01	0.03	0.02	0.02	0.02	0.02	10.05%

© 2018 Li Z et al. *JAMA Network Open.*

WHO region	Recipient country	Adolescent-targeted DAAH per adolescent (US$)													Annual growth rate (%)
		**2003**	**2004**	**2005**	**2006**	**2007**	**2008**	**2009**	**2010**	**2011**	**2012**	**2013**	**2014**	**2015**	
South-East Asia	Indonesia	0.01	0.01	0.02	0.01	0.02	0.03	0.02	0.20	0.17	0.12	0.07	0.02	0.01	3.70%
	Maldives	0.58	0.76	0.66	0.60	0.70	0.59	0.69	5.05	4.30	9.19	1.70	1.01	2.07	11.17%
	Myanmar	0.05	0.08	0.02	0.02	0.06	0.04	0.01	0.02	0.04	0.05	0.01	0.02	0.05	-0.83%
	Nepal	0.08	0.06	0.08	0.46	0.85	1.04	0.55	0.83	0.23	0.16	0.37	0.12	0.12	3.56%
	Sri Lanka	0.37	0.50	3.12	1.73	1.27	1.33	1.09	1.86	1.96	1.84	1.68	3.43	0.09	-11.10%
	Thailand	0.02	0.01	0.03	0.04	0.00	0.01	0.32	1.02	0.99	0.06	0.05	0.12	0.08	12.24%
	Timor-Leste	0.15	0.61	0.24	2.67	0.13	0.79	0.67	3.10	8.04	2.91	0.13	3.85	4.06	31.31%
Western Pacific	Cambodia	0.24	0.05	0.06	0.16	0.47	0.36	0.37	0.28	0.76	0.75	0.27	0.66	1.00	12.73%
	China	0.00	0.00	0.00	0.00	0.00	0.00	0.01	0.00	0.00	0.00	0.00	0.00	0.00	0.28%
	Fiji		1.70	2.07	1.83	2.35	1.70	0.01			3.51				9.48%
	Kiribati			63.61							6.10				-28.45%
	Lao	0.04	0.12	0.24	0.13	0.04	0.05	0.04	0.07	0.04	0.36	2.20	0.25	0.75	26.73%
	Malaysia	0.01	0.00	0.01	0.01	0.02	0.02	0.02	0.02	0.02	0.03	0.01	0.01	0.00	-3.86%
	Micronesia														
	Mongolia	0.90	0.42	0.13	0.23	0.01	0.16	3.48	1.66	0.98	2.65	2.43	2.60	2.65	9.37%
	Papua New Guinea	0.21	0.38	0.22	0.30	0.28	0.19	0.24	0.29	0.18	0.42	0.10	0.08	0.10	-5.54%
	Philippines	0.01	0.05	0.04	0.03	0.02	0.03	0.02	0.01	0.01	0.03	0.07	0.04	0.05	20.22%
	Samoa						0.87	0.20	0.03	0.00	5.99			0.29	-14.38%
	Solomon Islands	2.84	2.65		0.10						5.22				6.99%
	Tonga						0.88		0.61		13.32				97.12%
	Vanuatu								8.98	8.07	32.05	41.16	7.53	13.83	9.01%
	Viet Nam	0.03	0.07	0.07	0.03	0.06	0.05	0.04	0.18	0.13	0.07	0.01	0.02	0.09	10.50%

Note:

1. We didn't include unallocable or regional DAAH projects
2. All disbursement data were converted to constant 2015 US$
3. Annual growth rate=((end value/start value)^(1/periods) -1. For example, DAAH per adolescent in Bangladesh was $0.035 in 2003 (start value) and $0.312 in 2015 (end value) . The period is 2015-2003=12 years. The compound annual growth rate for Bangladesh is (0.312/0.035)^(1/12)-1=19.94%

© 2018 Li Z et al. *JAMA Network Open.*

eTable 19. DAAH (Adolescent Inclusive) per Adolescent (US$) by Recipient Country, 2003-2015

WHO region	Recipient country	Adolescent-targeted DAAH per adolescent (US$)													Annual growth rate (%)
		2003	2004	2005	2006	2007	2008	2009	2010	2011	2012	2013	2014	2015	
Africa	Algeria	0.09	0.10	0.09	0.10	0.12	0.14	0.10	0.09	0.18	0.20	0.18	0.52	0.66	18.65%
	Angola	1.70	2.22	3.98	2.71	3.05	3.34	2.53	4.05	2.72	3.57	4.52	2.39	2.65	3.75%
	Benin	3.35	5.47	4.74	5.08	3.84	7.12	14.01	14.35	12.24	7.31	8.01	9.52	8.92	8.50%
	Botswana	10.83	16.64	12.75	16.05	24.65	113.89	108.63	37.91	41.96	37.17	42.98	34.54	26.97	7.90%
	Burkina Faso	2.34	4.38	4.06	6.04	3.78	4.56	7.29	8.29	4.59	6.44	5.63	6.83	7.34	9.98%
	Burundi	5.72	6.75	11.72	8.33	5.16	7.49	28.38	10.03	9.64	9.42	11.03	12.27	10.24	4.97%
	Cabo Verde	19.97	28.42	32.22	23.42	24.99	29.77	31.84	46.19	60.78	57.84	68.83	72.90	67.55	10.69%
	Cameroon	3.19	4.98	5.07	6.96	3.33	2.03	4.32	2.23	4.02	4.30	6.27	4.33	6.48	6.07%
	Central African Republic	1.77	3.98	3.30	4.08	1.90	5.24	13.25	3.35	3.12	3.05	4.91	4.35	4.11	7.26%
	Chad	2.27	2.87	3.25	2.18	1.74	1.59	1.44	5.67	5.18	1.94	6.54	3.95	2.82	1.82%
	Comoros	5.84	5.80	2.25	2.22	2.00	2.38	2.50	12.05	12.95	12.71	11.11	11.22	8.51	3.20%
	Congo	3.92	3.75	5.36	6.65	7.42	6.03	5.00	12.88	11.79	5.33	6.39	3.87	5.43	2.75%
	Côte d'Ivoire	1.65	2.21	1.98	3.00	3.29	6.53	5.70	8.04	5.41	6.11	10.47	11.29	7.39	13.30%
	Democratic Republic of the Congo	0.87	1.15	1.87	1.73	1.80	3.62	3.74	5.40	5.06	6.98	4.79	4.66	5.53	16.71%
	Djibouti	3.29	3.27	10.33	8.15	11.17	11.04	22.34	11.43	12.96	11.59	9.52	10.49	9.31	9.04%
	Equatorial Guinea	3.19	3.72	5.88	10.97	7.77	16.04	11.35	14.28	6.17	3.49	0.89	3.63	4.99	3.80%

© 2018 Li Z et al. *JAMA Network Open.*

WHO region	Recipient country	Adolescent-targeted DAAH per adolescent (US$)													Annual growth rate (%)
		2003	2004	2005	2006	2007	2008	2009	2010	2011	2012	2013	2014	2015	
Africa	Eritrea	11.51	13.32	14.27	8.67	4.52	6.69	10.60	17.28	6.76	13.39	11.19	14.81	4.87	-6.91%
	Ethiopia	2.13	2.32	3.05	5.12	4.07	5.18	6.31	10.87	7.23	6.26	7.66	5.83	7.75	11.35%
	Gabon	2.01	3.56	7.01	6.30	4.53	3.34	9.32	7.32	5.73	3.19	5.39	4.18	3.02	3.45%
	Gambia	11.16	13.56	18.45	8.35	9.36	32.22	14.94	17.72	20.73	20.64	28.64	17.28	14.01	1.91%
	Ghana	1.74	3.26	4.49	3.24	4.22	5.19	13.00	9.11	8.55	10.45	15.21	12.44	15.54	19.99%
	Guinea	1.83	1.98	2.63	2.36	1.85	3.26	4.14	3.57	3.05	4.89	3.91	11.71	12.36	17.25%
	Guinea-Bissau	2.99	3.51	5.14	5.34	7.72	5.19	12.82	21.32	11.92	5.82	29.33	10.18	17.16	15.66%
	Kenya	4.92	6.12	5.77	7.31	8.54	10.12	15.98	16.65	15.26	17.40	19.85	17.49	16.30	10.49%
	Lesotho	4.37	4.21	7.41	7.34	11.53	17.04	35.83	37.25	41.44	39.90	51.45	22.75	18.34	12.70%
	Liberia	1.65	3.09	4.24	5.28	4.55	22.32	33.11	37.76	31.39	29.61	30.29	44.15	66.50	36.06%
	Madagascar	1.77	2.21	3.01	2.58	3.02	3.19	3.87	8.72	3.76	4.82	4.48	3.69	4.09	7.20%
	Malawi	6.33	8.14	9.32	10.08	13.61	13.99	15.43	15.22	20.37	23.15	22.90	23.18	30.22	13.92%
	Mali	2.49	3.65	4.84	9.94	8.28	8.98	10.41	13.33	9.00	11.51	13.96	14.87	13.35	15.01%
	Mauritania	7.62	8.22	3.74	24.76	5.79	6.92	5.20	1.77	2.26	2.27	2.58	2.92	2.68	-8.35%
	Mauritius	1.00	0.87	0.10	0.64	1.23	0.19	2.94	6.85	5.42	4.81	5.54	7.28	3.73	11.58%
	Mozambique	5.06	6.01	5.80	6.80	9.68	12.13	13.65	20.15	16.87	18.96	16.85	21.97	16.45	10.32%
	Namibia	8.23	13.16	17.88	36.42	43.42	41.03	89.62	78.95	44.54	73.64	51.43	46.02	33.54	12.42%
	Niger	1.44	3.23	3.67	4.53	3.81	4.30	5.50	3.83	3.75	3.17	4.38	8.09	6.91	13.95%

© 2018 Li Z et al. *JAMA Network Open.*

WHO region	Recipient country	Adolescent-targeted DAAH per adolescent (US$)													Annual growth rate (%)
		**2003**	**2004**	**2005**	**2006**	**2007**	**2008**	**2009**	**2010**	**2011**	**2012**	**2013**	**2014**	**2015**	
Africa	Nigeria	0.88	1.21	1.48	2.03	2.62	3.74	5.37	4.53	4.60	5.09	6.64	7.16	7.52	19.60%
	Rwanda	5.63	8.53	9.64	17.30	12.51	19.46	26.43	31.66	26.11	26.67	23.22	25.83	25.66	13.48%
	Sao Tome and Principe	35.33	31.07	45.27	34.75	31.96	25.98	14.49	18.83	51.69	40.22	66.53	58.34	47.58	2.51%
	Senegal	5.03	8.49	9.25	22.62	6.69	11.00	13.13	11.55	9.89	9.02	8.51	10.90	13.15	8.35%
	Seychelles	4.56	12.95	10.64	0.90	1.20	1.58	0.81	10.09	17.30	43.01	10.03	3.95	10.96	7.57%
	Sierra Leone	2.55	6.42	7.75	9.78	13.87	9.89	13.41	18.17	15.89	20.39	19.99	30.25	32.54	23.64%
	Somalia	1.07	1.42	1.57	1.87	2.92	2.16	4.23	5.80	11.27	12.79	10.45	9.74	11.24	21.63%
	South Africa	1.78	2.61	3.44	3.99	6.26	9.12	14.01	13.82	13.29	14.52	13.23	14.19	11.10	16.50%
	South Sudan									7.38	12.29	14.41	18.36	30.25	42.27%
	Sudan	0.44	1.15	2.42	2.22	2.23	5.29	5.90	8.02	5.28	4.46	3.98	1.48	2.12	14.09%
	Swaziland	6.89	3.90	19.71	13.15	16.56	17.90	37.35	41.40	45.31	40.11	46.08	34.50	36.66	14.95%
	Togo	1.58	2.25	3.35	2.89	4.46	3.71	10.30	7.47	7.50	3.53	9.41	8.53	6.01	11.75%
	Uganda	3.90	5.58	7.45	7.27	8.93	8.62	27.25	12.37	11.40	15.06	14.55	14.15	14.33	11.47%
	United Republic of Tanzania	5.92	4.71	4.96	9.11	6.63	9.30	11.18	14.87	12.86	14.16	21.18	18.84	13.02	6.79%
	Zambia	5.83	9.44	13.51	11.67	15.11	23.71	24.05	20.32	25.45	28.89	36.65	27.29	21.44	11.45%
	Zimbabwe	3.09	2.86	5.58	5.26	7.87	5.33	8.68	12.78	17.13	42.17	26.49	22.46	26.68	19.67%

© 2018 Li Z et al. *JAMA Network Open.*

WHO region	Recipient country	Adolescent-targeted DAAH per adolescent (US$)													Annual growth rate (%)
		2003	2004	2005	2006	2007	2008	2009	2010	2011	2012	2013	2014	2015	
Americas	Argentina	14.02	3.13	2.21	2.03	4.34	2.82	5.20	9.91	19.83	18.02	21.56	17.47	13.77	-0.15%
	Belize	4.08	5.11	20.47	18.72	20.62	15.06	30.44	18.90	10.27	21.78	26.22	2.53	31.79	18.67%
	Bolivia	5.58	5.52	4.79	6.72	7.17	8.78	9.99	13.63	12.26	8.72	9.28	6.21	10.77	5.63%
	Brazil	0.32	0.57	1.26	1.33	0.96	0.62	0.30	0.66	0.65	0.82	1.19	0.85	0.38	1.48%
	Chile	0.37	0.80	0.95	0.43	0.80	0.31	0.15	0.29	0.26	0.21	0.40	0.18	0.15	-7.13%
	Colombia	1.94	2.80	4.25	4.63	5.10	4.47	4.36	15.83	9.29	3.39	3.37	2.06	2.11	0.70%
	Costa Rica	0.58	0.85	1.74	1.92	2.29	2.20	3.80	3.41	1.30	0.86	0.87	0.49	0.65	0.95%
	Cuba	1.66	1.66	1.03	1.17	1.92	1.57	1.15	5.42	4.33	4.07	4.65	1.65	4.83	9.31%
	Dominican Republic	1.61	3.38	4.78	5.46	7.75	8.27	13.09	15.46	12.16	9.25	25.86	7.74	12.05	18.28%
	Ecuador	1.77	1.22	2.45	2.47	2.53	5.27	3.71	2.54	2.65	1.55	2.37	2.13	1.64	-0.63%
	El Salvador	1.65	2.18	10.02	6.10	6.31	9.84	12.85	14.47	10.88	9.82	10.17	8.14	9.59	15.80%
	Grenada	2.45	1.22	9.18	5.28	7.68	4.38	16.55	4.87	14.94	5.91	3.86	2.26	3.80	3.72%
	Guatemala	2.19	1.74	2.22	2.56	3.66	7.30	8.22	11.33	10.58	6.95	6.33	6.81	5.39	7.81%
	Guyana	19.32	24.46	37.18	67.01	58.16	44.67	67.50	44.86	44.52	28.45	21.94	17.73	14.50	-2.36%
	Haiti	3.53	4.46	7.38	12.25	17.66	21.02	31.62	25.05	28.62	25.03	26.45	24.62	27.35	18.61%
	Honduras	4.99	3.96	4.69	5.93	6.82	11.98	16.22	11.43	12.71	10.20	19.85	7.30	14.87	9.53%
	Jamaica	9.07	8.73	18.29	19.59	21.65	19.92	18.65	10.19	12.42	9.18	10.93	17.99	25.63	9.04%
	Mexico	0.10	0.09	0.09	1.11	0.81	0.19	0.16	0.36	0.27	0.29	0.21	0.13	0.27	8.49%

© 2018 Li Z et al. *JAMA Network Open.*

WHO region	Recipient country	Adolescent-targeted DAAH per adolescent (US$)													Annual growth rate (%)
		2003	2004	2005	2006	2007	2008	2009	2010	2011	2012	2013	2014	2015	
America	Nicaragua	5.89	4.76	7.90	8.47	12.56	12.56	22.18	17.95	15.49	14.95	14.03	11.85	19.26	10.37%
	Panama	0.12	0.58	3.33	3.02	3.76	3.60	6.64	3.78	3.26	6.40	5.17	0.90	1.49	23.11%
	Paraguay	2.37	1.67	2.97	3.22	4.39	4.69	7.58	8.69	5.35	5.78	5.32	2.06	2.70	1.10%
	Peru	1.13	1.82	2.23	1.98	2.54	4.37	7.00	4.87	3.26	3.21	2.91	2.08	6.57	15.78%
	Saint Vincent and the Grenadines	5.96	3.43	4.24	4.54	2.59	10.83	13.37	15.44	92.97	61.41	35.89	15.47	13.26	6.90%
	Suriname	0.71	1.39	6.12	5.84	70.00	4.21	11.27	11.05	4.57	0.77	6.48	15.92	35.05	38.39%
	Uruguay	19.97	7.48	14.94	12.17	14.67	15.47	38.07	21.76	24.10	5.97	9.25	6.17	10.18	-5.46%
	Venezuela (Bolivarian Republic of)	0.53	0.20	0.31	0.21	1.22	1.35	11.28	9.71	7.67	13.61	12.18	11.66	6.50	23.27%
Eastern Mediterranean	Afghanistan	0.07	0.08	0.44	0.18	0.16	0.20	0.18	0.67	1.38	1.99	3.52	1.81	2.61	35.32%
	Egypt	0.74	0.45	3.97	0.85	2.02	9.64	23.48	24.86	19.32	11.41	10.11	16.23	12.81	26.77%
	Iran (Islamic Republic of)	1.48	1.10	1.14	0.77	0.74	3.30	4.33	1.49	1.05	1.38	1.51	1.68	1.27	-1.25%
	Iraq	0.04	0.21	2.43	0.44	0.22	0.36	0.22	0.22	0.28	0.37	0.30	0.21	0.22	16.35%
	Jordan	0.28	0.27	11.94	7.71	7.03	3.45	3.84	2.24	0.88	1.54	1.03	1.69	2.73	20.99%
	Lebanon	10.01	7.23	1.30	2.98	3.30	10.06	20.69	33.31	32.49	26.96	30.74	34.78	19.79	5.84%
	Libya	5.02	5.20	11.26	13.53	22.25	21.34	21.24	19.10	16.82	19.28	17.99	27.51	21.14	12.72%
	Morocco			0.00	0.07	0.21	3.48	2.33	0.08	1.40	2.72	1.05	1.63	0.75	29.44%
	Pakistan	0.92	0.76	1.01	1.06	4.57	1.89	2.55	1.76	1.95	2.71	3.28	5.49	1.76	5.61%
	Syrian Arab Republic	0.39	0.32	0.58	0.81	2.56	0.63	3.97	2.74	2.32	2.24	2.51	2.02	2.68	17.35%
	Tunisia	0.18	0.28	0.15	0.16	0.32	0.56	0.97	1.09	1.25	0.26	3.56	6.38	7.12	35.60%
	Yemen	0.26	0.47	0.95	1.19	0.95	0.78	1.88	2.78	4.74	8.30	7.85	6.78	6.12	30.15%

© 2018 Li Z et al. *JAMA Network Open.*

WHO region	Recipient country	Adolescent-targeted DAAH per adolescent (US$)													Annual growth rate (%)
		**2003**	**2004**	**2005**	**2006**	**2007**	**2008**	**2009**	**2010**	**2011**	**2012**	**2013**	**2014**	**2015**	
Europe	Albania	4.44	4.05	5.14	4.37	5.89	8.55	10.01	10.85	12.61	7.87	5.69	10.03	11.73	8.42%
	Armenia	1.35	1.36	5.09	6.17	9.67	15.41	22.19	13.31	14.48	12.18	13.45	10.04	11.93	19.91%
	Azerbaijan	0.27	0.23	1.09	1.70	1.72	2.01	2.77	2.85	3.63	2.14	2.71	2.11	0.74	8.73%
	Belarus			0.89	1.69	1.18	2.38	2.97	6.15	7.24	7.25	4.10	5.24	5.00	18.89%
	Bosnia and Herzegovina	7.28	4.60	3.26	11.18	12.39	11.04	15.88	26.45	23.44	25.76	24.14	34.02	22.15	9.71%
	Georgia	1.39	1.43	4.59	5.69	10.61	12.18	18.08	12.88	22.04	23.42	19.08	19.04	18.85	24.25%
	Kazakhstan	0.65	0.89	1.36	2.25	1.55	2.85	3.33	2.35	1.93	1.78	1.12	0.93	1.04	3.97%
	Kyrgyzstan	2.88	1.85	3.93	3.28	4.81	6.76	8.91	9.02	11.26	11.41	11.16	9.75	12.01	12.65%
	Mayotte	14.63	32.88	2.49	1.71	19.34	46.25	120.63	110.28						33.45%
	Moldova	1.97	3.82	7.52	7.66	14.19	10.13	12.91	17.01	14.52	10.59	14.78	18.11	23.24	22.84%
	Montenegro				5.95	9.56	17.87	3.55	8.55	7.45	7.23	8.98	7.14	9.12	4.86%
	Serbia	2.41	2.58	1.35	2.51	2.47	5.53	8.17	9.27	24.70	8.74	15.36	14.34	4.61	5.57%
	Tajikistan	1.35	1.67	1.43	0.92	1.85	2.76	2.25	4.97	5.11	7.19	7.33	6.68	3.70	8.73%
	The former Yugoslav Republic of Macedonia	3.30	7.44	6.24	10.10	10.19	25.50	24.24	10.21	13.92	14.46	24.53	12.36	20.44	16.41%
	Turkey	0.24	0.18	0.22	0.90	0.42	0.53	0.64	0.73	2.04	0.07	0.21	0.21	0.28	1.47%
	Turkmenistan	0.30	0.17	1.06	0.93	1.23	1.52	1.78	0.72	1.48	0.72	1.32	1.20	0.99	10.34%
	Ukraine			1.69	2.78	3.90	3.27	6.00	4.19	5.34	4.52	3.98	26.72	28.46	32.59%
	Uzbekistan	0.39	0.35	0.60	0.51	0.67	0.80	1.31	1.59	1.34	1.89	0.54	0.90	1.34	10.80%
South-East Asia	Bangladesh	0.97	0.92	0.94	1.32	0.88	1.15	1.80	1.52	1.59	1.23	2.71	2.82	2.44	8.01%
	Bhutan	5.76	3.69	4.12	7.33	5.81	6.10	6.64	6.98	7.38	8.57	10.88	15.99	15.26	8.46%
	Democratic People's Republic of Korea	0.57	0.17	0.20	0.11	0.12	0.15	0.22	0.91	0.72	0.59	0.57	1.05	0.69	1.71%
	India	0.32	0.45	0.62	0.42	0.47	0.46	0.61	0.64	0.76	0.53	0.58	0.45	0.66	6.24%

© 2018 Li Z et al. *JAMA Network Open.*

WHO region	Recipient country	Adolescent-targeted DAAH per adolescent (US$)													Annual growth rate (%)
		2003	2004	2005	2006	2007	2008	2009	2010	2011	2012	2013	2014	2015	
South-East Asia	Indonesia	0.54	0.87	0.59	0.75	0.67	1.08	1.70	1.52	1.17	1.06	0.94	0.62	0.66	1.74%
	Maldives	4.68	4.43	2.67	1.10	7.08	4.00	2.94	9.31	15.08	18.20	7.00	3.93	6.57	2.87%
	Myanmar	0.95	0.61	0.83	0.52	1.19	1.36	1.44	2.12	1.49	2.84	3.89	4.66	3.74	12.13%
	Nepal	3.95	3.15	2.33	2.66	2.38	4.52	4.44	8.06	6.87	4.93	4.60	4.96	5.75	3.18%
	Sri Lanka	1.22	1.67	6.59	6.89	5.58	6.04	10.37	7.91	6.59	4.47	3.75	10.44	4.08	10.62%
	Thailand	0.41	0.74	0.76	1.61	1.23	1.19	1.55	2.19	2.98	1.47	2.46	1.99	0.74	5.00%
	Timor-Leste	5.51	6.20	9.22	10.90	13.18	32.28	42.32	50.62	48.28	41.04	20.75	16.98	16.48	9.56%
Western Pacific	Cambodia	3.71	3.29	5.31	5.12	6.16	7.14	11.18	10.22	9.44	7.32	8.77	10.26	11.17	9.63%
	China	0.09	0.16	0.12	0.15	0.20	0.17	0.26	0.20	0.18	0.23	0.12	0.10	0.11	1.29%
	Fiji	7.91	6.73	9.61	10.14	11.01	9.45	10.95	6.48	4.29	17.87	6.31	7.35	14.10	4.93%
	Kiribati	1.82	1.44	99.61	39.56	37.93	32.90	34.15	39.36	29.22	150.40	101.39	71.50	215.01	48.84%
	Lao	1.83	1.77	3.46	2.89	3.85	4.04	3.21	3.84	5.01	4.72	7.83	5.33	8.52	13.69%
	Malaysia	0.03	0.05	0.02	0.01	0.06	0.02	0.02	0.02	0.11	0.17	0.22	0.20	0.14	13.04%
	Micronesia	11.05	1.08	10.04	9.18	8.71	8.56	10.86	1.15	1.68	1.21	1.81	2.18	2.08	-13.00%
	Mongolia	2.78	1.63	2.69	2.99	3.98	5.00	8.21	8.16	8.71	11.55	11.98	12.80	10.56	11.75%
	Papua New Guinea	3.94	4.27	4.03	4.77	4.53	4.93	17.14	6.61	12.36	15.03	19.71	15.11	21.40	15.13%
	Philippines	0.71	0.67	1.24	1.22	1.27	1.26	1.58	2.14	1.71	1.71	2.03	2.05	1.95	8.80%
	Samoa	10.66	3.34	22.57	27.65	21.38	19.35	22.98	76.19	20.12	77.74	57.45	17.20	15.81	3.34%
	Solomon Islands	6.28	15.51	15.41	0.59	2.52	20.86	23.61	12.32	10.52	45.13	48.43	24.43	26.46	12.74%
	Tonga	21.60	10.05	97.58	51.82	37.53	45.29	61.55	36.14	98.84	98.34	5.48	6.49	15.69	-2.63%
	Vanuatu	21.00	9.26	30.91	29.35	32.23	40.68	47.25	18.06	12.77	67.16	112.11	123.99	140.83	17.18%
	Viet Nam	1.05	0.97	1.16	1.38	1.50	1.76	2.57	2.66	2.86	2.82	2.55	2.05	1.83	4.69%

Note:

1. We didn’t include unallocable or regional DAAH projects
2. All disbursement data were converted to constant 2015 US$
3. Annual growth rate=((end value/start value)^(1/periods) -1. For example, DAAH per adolescent in Vanuatu was $21.00 in 2003 (start value) and $140.83 in 2015 (end value). The period is 2015-2003=12 years. The compound annual growth rate for Vanuatu is (140.83/21.00)^(1/12)-1=17.18%

© 2018 Li Z et al. *JAMA Network Open.*

eFigure 5. Annual Growth Rate in DAAH (Adolescent Inclusive) per Adolescent, 2003-2015 (in percentage)

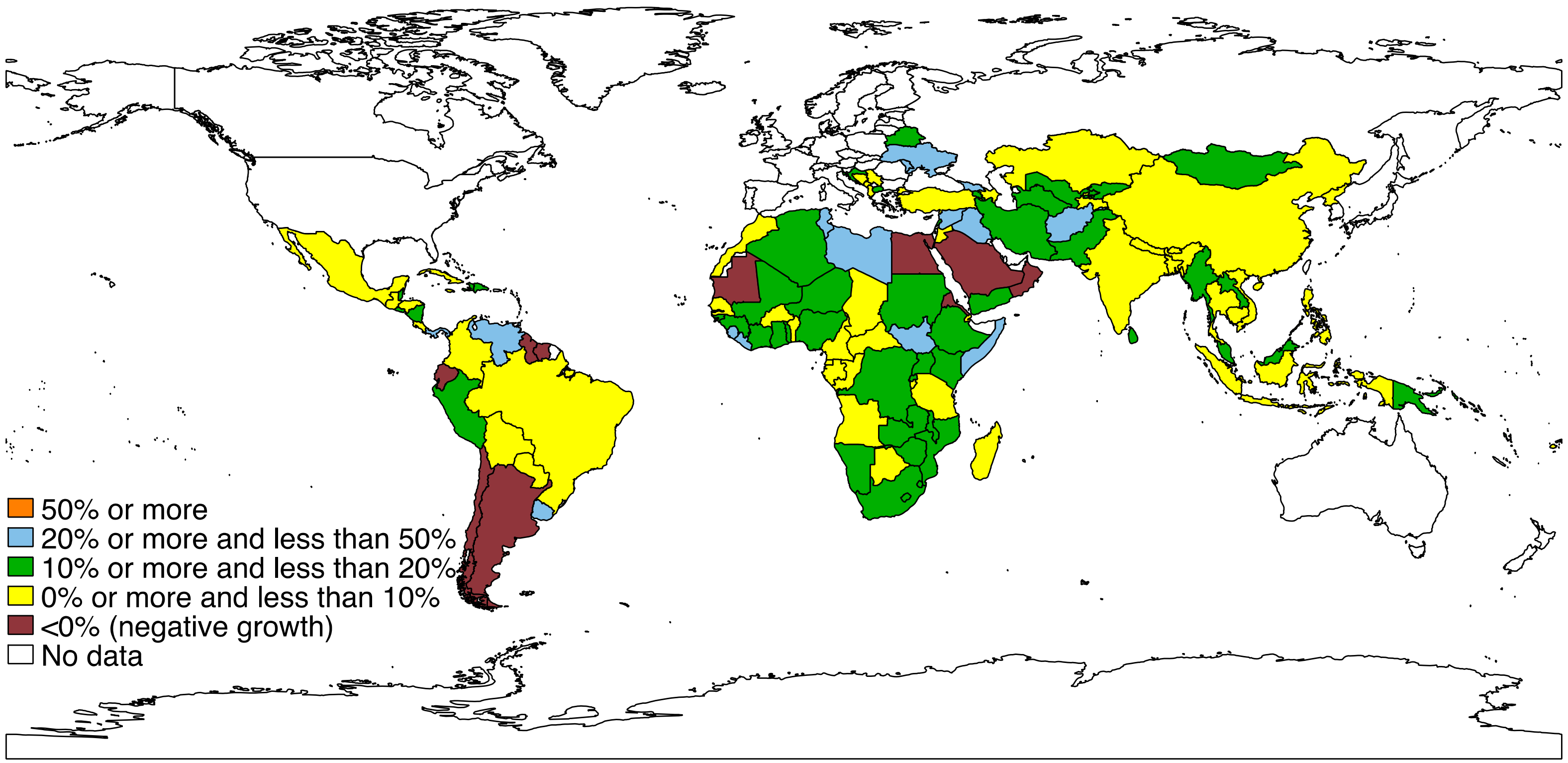

Note:

1. We didn't include unallocable or regional DAAH projects
2. All disbursement data were converted to constant 2015 US$
3. Annual growth rate=((end value/start value)^(1/periods) -1.

© 2018 Li Z et al. *JAMA Network Open.*

eReferences

1. Institute for Health Metrics and Evaluation. GBD Results Tool [Internet]. [cited 2018 Jan 11]. Available from: http://ghdx.healthdata.org/gbd-results-tool
2. ICD. 2018 ICD-10-CM Codes [Internet]. [cited 2018 Jan 11]. Available from: http://www.icd10data.com/ICD10CM/Codes
3. World Health Organization. WHO | World report on child injury prevention. WHO [Internet]. 2015 [cited 2017 Dec 25]; Available from: http://www.who.int/violence_injury_prevention/child/injury/world_report/en/
4. Centers for Disease Control and Prevention. Guidelines for prevention and treatment opportunistic infections in HIV-infected adults and adolescents; recommendations from CDC, the National Institutes of Health, and the HIV Medicine Association/Infectious Diseases Society of America [Internet]. CDC Stacks Public Health Publications. 2009 [cited 2017 Nov 1]. Available from: https://stacks.cdc.gov/view/cdc/5236
5. World Health Organization. Guidelines for prevention and treatment opportunistic infections in HIV-infected adults and adolescents; recommendations from CDC, the National Institutes of Health, and the HIV Medicine Association/Infectious Diseases Society of America [Internet]. 2012 [cited 2017 Nov 1]. Available from: http://www.who.int/hiv/en/
6. World Health Organization. Psychosocial Support [Internet]. WHO. World Health Organization; 2006 [cited 2017 Nov 1]. Available from: http://www.who.int/hiv/topics/psychosocial/support/en/
7. Bermejo F, García-López S. A guide to diagnosis of iron deficiency and iron deficiency anemia in digestive diseases. World J Gastroenterol. 2009 Oct 7;15(37):4638–43.
8. Camaschella C. Iron-Deficiency Anemia. Longo DL, editor. N Engl J Med. 2015 May 7;372(19):1832–43.
9. Hawton K, Saunders KE, O'Connor RC. Self-harm and suicide in adolescents. Lancet. 2012 Jun;379(9834):2373–82.
10. GoodTherapy website. Therapy for Self Harm, Therapist for Self Harm Issues [Internet]. [cited 2018 Jan 12]. Available from: https://www.goodtherapy.org/learn-about-therapy/issues/self-harm
11. World Health Organization. WHO | Assessment for self harm/suicide in persons with priority mental, neurological and substance use disorders. WHO [Internet]. 2016 [cited 2018 Jan 12]; Available from: http://www.who.int/mental_health/mhgap/evidence/suicide/q1/en/
12. World Health Organization. WHO | Youth violence [Internet]. WHO. World Health Organization; 2016 [cited 2017 Aug 9]. Available from: http://who.int/violence_injury_prevention/violence/youth/en/
13. Krug EG, Mercy JA, Dahlberg LL, Zwi AB. The world report on violence and health. Lancet. 2002 Oct 5;360(9339):1083–8.
14. Resnick MD, Bearman PS, Blum RW, Bauman KE, Harris KM, Jones J, et al. Protecting Adolescents From Harm. JAMA. 1997 Sep 10;278(10):823.
15. Kessler RC, Berglund P, Demler O, Jin R, Koretz D, Merikangas KR, et al. The Epidemiology of Major Depressive Disorder. JAMA. 2003 Jun 18;289(23):3095.
16. Cuijpers P, Beekman ATF, Reynolds CF, III. Preventing depression: a global priority. JAMA. 2012 Mar 14;307(10):1033–4.
17. Mayo Clinic. Depression (major depressive disorder) - Diagnosis and treatment - Mayo Clinic [Internet]. [cited 2018 Jan 12]. Available from: https://www.mayoclinic.org/diseases-conditions/depression/diagnosis-treatment/drc-20356013
18. MacGregor AJ, Andrew T, Sambrook PN, Spector TD. Structural, psychological, and genetic influences on low back and neck pain: A study of adult female twins. Arthritis Care Res (Hoboken). 2004 Apr 15;51(2):160–7.
19. Strine TW, Hootman JM. US national prevalence and correlates of low back and neck pain among adults. Arthritis Rheum. 2007 May 15;57(4):656–65.
20. Mayo Clinic. Diarrhea - Diagnosis and treatment - Mayo Clinic [Internet]. [cited 2018 Jan 12]. Available from: https://www.mayoclinic.org/diseases-conditions/diarrhea/diagnosis-treatment/drc-20352246
21. World Health Organization. WHO | Water, sanitation and hygiene interventions and the prevention of diarrhoea. WHO [Internet]. 2015 [cited 2017 Dec 25]; Available from: http://www.who.int/elena/titles/bbc/wsh_diarrhoea/en/
22. Trunz BB, Fine P, Dye C. Effect of BCG vaccination on childhood tuberculous meningitis and miliary tuberculosis worldwide: a meta-analysis and assessment of cost-effectiveness. Lancet. 2006;367(9517):1173–80.
23. Colditz GA, Brewer TF, Berkey CS, Wilson ME, Burdick E, Fineberg H V., et al. Efficacy of BCG Vaccine in the Prevention of Tuberculosis. JAMA. 1994 Mar 2;271(9):698.
24. Lienhardt C, Vernon A, Raviglione MC. New drugs and new regimens for the treatment of tuberculosis: review of the drug development pipeline and implications for national programmes. Curr Opin Pulm Med. 2010 Mar;1.
25. Mayo Clinic. Tuberculosis - Diagnosis and treatment - Mayo Clinic [Internet]. [cited 2018 Jan 12]. Available from: https://www.mayoclinic.org/diseases-conditions/tuberculosis/diagnosis-treatment/drc-20351256
26. World Health Organization. TB Prevention, Diagnosis and Treatment. [cited 2018 Jan 12]; Available from: http://www.who.int/tb/challenges/hiv/07_tb_prevention_diagnosis_and_treatment_eng.pdf
27. American Academy of Pediatrics Committee on Injury, Violence, and Poison Prevention, Violence and PP, Wentz KR, Gore EJ, Copass MK. Prevention of drowning. Pediatrics. 2010 Jul 1;126(1):178–85.

© 2018 Li Z et al. *JAMA Network Open.*
